# Network meta-analysis of different electrical stimulation therapies for lower limb functional rehabilitation in stroke patients

**DOI:** 10.3389/fneur.2025.1682671

**Published:** 2026-01-12

**Authors:** Juyao Liu, Fukun Zeng, Zhuojun Peng, Shuizhen Wen, Ding Liu, Seng Tang, Huaxin Su

**Affiliations:** The First Affiliated Hospital of Hunan College of Traditional Chinese Medicine, Hunan Province Directly Affiliated TCM Hospital, Zhuzhou, China

**Keywords:** electrical stimulation, lower limb function, network meta-analysis, randomized controlled trials, stroke

## Abstract

**Introduction:**

Electrical stimulation is widely applied in the rehabilitation of post-stroke lower limb dysfunction; however, its comparative efficacy and safety across different modalities remain unclear. Substantial heterogeneity among electrical stimulation techniques limits evidence-based clinical decision-making, highlighting the need for a comprehensive comparative evaluation.

**Methods:**

A comprehensive literature search was conducted across seven databases (CNKI, Wanfang, VIP, PubMed, Embase, Web of Science, and the Cochrane Library) from inception to June 2025. Randomized controlled trials evaluating electrical stimulation interventions for post-stroke lower limb dysfunction were included. Methodological quality was assessed using the Cochrane Risk of Bias 2.0 tool. Network meta-analysis was performed using Stata 18.0 and R 4.3.2, and treatment rankings were estimated based on surface under the cumulative ranking curve (SUCRA) probabilities.

**Results:**

A total of 81 randomized controlled trials involving 6,147 patients and 24 intervention strategies were included. Network meta-analysis demonstrated that: (1) For lower limb motor function (Fugl–Meyer Assessment, lower extremity), electromyography-triggered functional electrical stimulation combined with conventional functional electrical stimulation ranked highest (SUCRA = 89.0%), whereas conventional therapy ranked lowest (SUCRA = 4.3%). (2) For balance ability (Berg Balance Scale), multi-channel functional electrical stimulation showed the greatest efficacy (SUCRA = 85.6%), compared with conventional therapy (SUCRA = 4.2%). (3) For activities of daily living (Modified Barthel Index), closed-loop neuromuscular electrical stimulation was most effective (SUCRA = 71.9%), while conventional therapy ranked lowest (SUCRA = 3.0%). (4) For walking speed (10-Meter Walk Test), low-frequency electrical stimulation demonstrated superior efficacy (SUCRA = 66.2%) compared with neuromuscular electrical stimulation (SUCRA = 35.6%). (5) For functional ambulation (Functional Ambulation Category), transcranial direct current stimulation achieved the highest ranking (SUCRA = 99.7%).

**Conclusion:**

Different electrical stimulation modalities provide domain-specific benefits in post-stroke lower limb rehabilitation. Tailored selection of stimulation techniques may optimize functional recovery. Nevertheless, the overall evidence remains limited, and further large-scale, high-quality randomized trials are required to confirm these findings and elucidate underlying neuroregulatory mechanisms.

**Systematic review registration:**

PROSPERO, identifier CRD420251087696.

## 1 Introduction

Stroke is defined as a severe cerebrovascular disorder characterized by abrupt cessation of cerebral perfusion, resulting in neuronal injury or demise, and presenting with a clinical spectrum spanning mild functional deficits to profound disability (1). Globally, approximately 12.2 million new stroke cases, 101 million prevalent stroke cases, and 6.55 million stroke-related deaths were recorded (2). With the global prevalence of stroke continuing to rise, about 55–75% of stroke survivors experience motor dysfunction, among which lower limb dysfunction accounts for a significant proportion (3, 4). Stroke patients manifest heterogeneous degrees of lower extremity motor impairment, principally characterized by diminished muscular strength, spasticity (predominantly in periarticular musculature of the hip/knee/ankle complexes), and aberrant gait kinematics. Furthermore, the non-paretic limb demonstrates concomitant limitations including restricted knee joint range of motion, compromised muscular strength, and reduced movement velocity (5, 6). Such dysfunction substantially elevates fall susceptibility among stroke survivors, detrimentally affecting ambulatory capacity, activities of daily living (ADL), and quality of life while generating significant familial and societal caregiver burdens. Thus, lower extremity functional rehabilitation constitutes a critical therapeutic target for optimizing post-stroke prognosis.

The main treatment methods for lower limb dysfunction after stroke include conventional rehabilitation training and electrical stimulation. Conventional rehabilitation training (e.g., basic motor exercises, balance training, gait training) exhibits moderate efficacy in ameliorating limb function in stroke patients with lower limb dysfunction (7, 8) However, this regimen is constrained by protracted duration and protocol homogeneity, frequently inducing patient fatigue and consequently diminishing treatment adherence. Current clinical electrical stimulation strategies show a trend toward diversification, including Functional Electrical Stimulation (FES), Transcutaneous Electrical Nerve Stimulation (TENS), Transcranial Direct Current Stimulation (tDCS), and Neuromuscular Electrical Stimulation (NMES). Electrical stimulation induces long-term synaptic plasticity alterations by directly modulating cerebral activity through electrical currents or magnetic fields applied to brain tissue (9). For example, FES activates motor neurons through emulation of physiological movement patterns, potentiating corticospinal tract excitability to enhance motor control (10, 11). The tDCS facilitates neuroplasticity by bidirectionally modulating cortical excitability—anodal stimulation augments excitability, while cathodal stimulation suppresses pathological hyperexcitability (12). Neuromuscular Electrical Stimulation optimizes muscle contraction force at 20-30 Hz frequencies to strengthen lower limb muscle power and motor function (13, 14). Nevertheless, current clinical research on post-stroke lower limb dysfunction primarily focuses on analyzing the efficacy of single-mode electrical stimulation combined with conventional rehabilitation, and there remains no unified conclusion regarding the comparative effects of different stimulation modalities.

As an emerging statistical methodology in evidence-based medicine, Network Meta-Analysis (NMA) concurrently synthesizes direct and indirect evidence, facilitating simultaneous evaluation of multiple interventions within a single analysis—including those lacking head-to-head comparisons in original studies—thereby generating clinically informative evidence for decision-making. Moreover, NMA enables hierarchical ranking of interventions according to their comparative efficacy and posterior probability of being the optimal treatment. This research implemented rigorous screening and synthesis of published clinical literature, utilizing NMA to comparatively evaluate the effects of diverse electrical stimulation modalities on lower limb motor function and ADL in stroke patients, with the objectives of furnishing robust evidence-based support for clinical practice, informing decision-making, and establishing an evidence-based reference framework.

## 2 Methods

This meta-analysis was conducted adhering to the Preferred Reporting Items for Systematic Reviews and Meta-Analyses extension for Network Meta-Analysis (PRISMA-NMA) statement. In Supplementary material 1, a list of PRISMA was provided. Furthermore, a study protocol was prospectively registered on the PROSPERO international prospective register of systematic reviews (Registration ID: CRD420251087696).

### 2.1 Search strategy

The search strategy was executed across seven bibliographic databases: CNKI, Wanfang Data, VIP, PubMed, Cochrane Library, Embase, and Web of Science. Search methodology incorporated a combination of controlled Medical Subject Headings (MeSH) vocabulary and unrestricted free-text terminology. The specific search strategies for each database are detailed in Supplementary material 2. Inclusion criteria restricted evidence sources to peer-reviewed publications exclusively in the Chinese or English languages.

### 2.2 Eligibility criteria

#### 2.2.1 Inclusion criteria

① Study Type and Language: Randomized controlled trials (RCTs) on different electromagnetic stimulation therapies for post-stroke lower limb dysfunction, limited to Chinese and English publications. ② Patients with stroke (brain bleed or clot confirmed by CT/MRI scans) showing leg mobility issues. Must be adults (>18 years old)—no restrictions on gender or how long since stroke occurred. ③ Control group: Standard stroke care (meds/rehab) or placebo stimulation. Intervention group: Control treatment plus active stimulations (e.g., nerve/muscle stimulation, brain stimulation techniques) or direct comparisons between stimulation types. ④ Outcome Measures: Fugl-Meyer Assessment for Lower Extremity (FMA-L), Berg Balance Scale (BBS), Modified Barthel Index (MBI), 10-Meter Walk Test (10MWT), Functional Ambulation Category (FAC).

#### 2.2.2 Exclusion criteria

①Non-randomized controlled trials. ② Publications with inaccessible full-text documents. ③ Literature published in languages other than Chinese or English. ④ Redundant publications (identical studies appearing in multiple sources). ⑥ Systematic reviews, animal experimentation, case reports, and conference proceedings.

### 2.3 Study selection

Following duplicate removal via EndNote X9 (2025 release), dual investigators independently conducted literature screening. Primary filtering utilized titles/keywords/abstracts, succeeded by full-text assessment for data extraction and cross-verification to ascertain eligible studies. A third reviewer audited the database, facilitating consensus resolution for any data extraction discrepancies. Extracted parameters comprised: primary author, publication year, sample size (intervention/control), mean age (intervention/control), therapeutic protocols (intervention/control), treatment duration, and outcome metrics.

### 2.4 Risk of bias and evidence quality assessment

The methodological risk of bias in included studies was appraised utilizing the Cochrane Risk of Bias Tool 2.0 (RoB 2.0). Per the Cochrane Handbook for Systematic Reviews of Interventions, dual reviewers independently conducted methodological quality assessments. Discordances in evaluations were reconciled via consensus deliberations or arbitration by an independent third reviewer. Assessments for individual trials prioritized five critical domains: ① Bias from randomization procedures. ② Bias from intervention deviations. ③ Bias from missing outcome data. ④ Bias in outcome measurement. ⑤ Bias in selective result reporting.

### 2.5 Statistical analysis

In this study, we conducted a Bayesian NMA using the gemtc package in R 4.3.2. Within the Bayesian framework, we specified 2,000 burn-in iterations followed by 50,000 sampling iterations. Convergence of the Markov chains was assessed through trace plots, posterior density plots, and the Brooks–Gelman–Rubin diagnostic, ensuring satisfactory mixing and model stability. Given the anticipated clinical diversity among the included trials—particularly regarding stimulation protocols, rehabilitation regimens, and participant characteristics—we prespecified a random-effects model for the NMA. To complement the network analysis and examine heterogeneity across direct comparisons, we additionally performed pairwise meta-analyses in Stata 18.0 where head-to-head data were available. For continuous outcomes, effect sizes were summarized using mean differences (MD) with corresponding 95% confidence intervals (95% CI). Statistical heterogeneity was quantified using the I^2^ statistic and its associated *p* value: a fixed-effect model was applied when *I^2^* < 50% and *p* > 0.1, whereas a random-effects model was adopted when *I^2^* ≥ 50% or *p* < 0.1. When appropriate, subgroup or sensitivity analyses were conducted to explore potential sources of heterogeneity. To evaluate the robustness of the findings, sensitivity analyses were performed for all key outcomes. For endpoints with at least 10 contributing studies, potential publication bias was examined using funnel plots and the Egger regression test (Stata 18.0). Symmetrical funnel plots together with an Egger test *p* > 0.05 were interpreted as indicating no significant publication bias.

## 3 Result

### 3.1 Research selection

**Figure 1:**
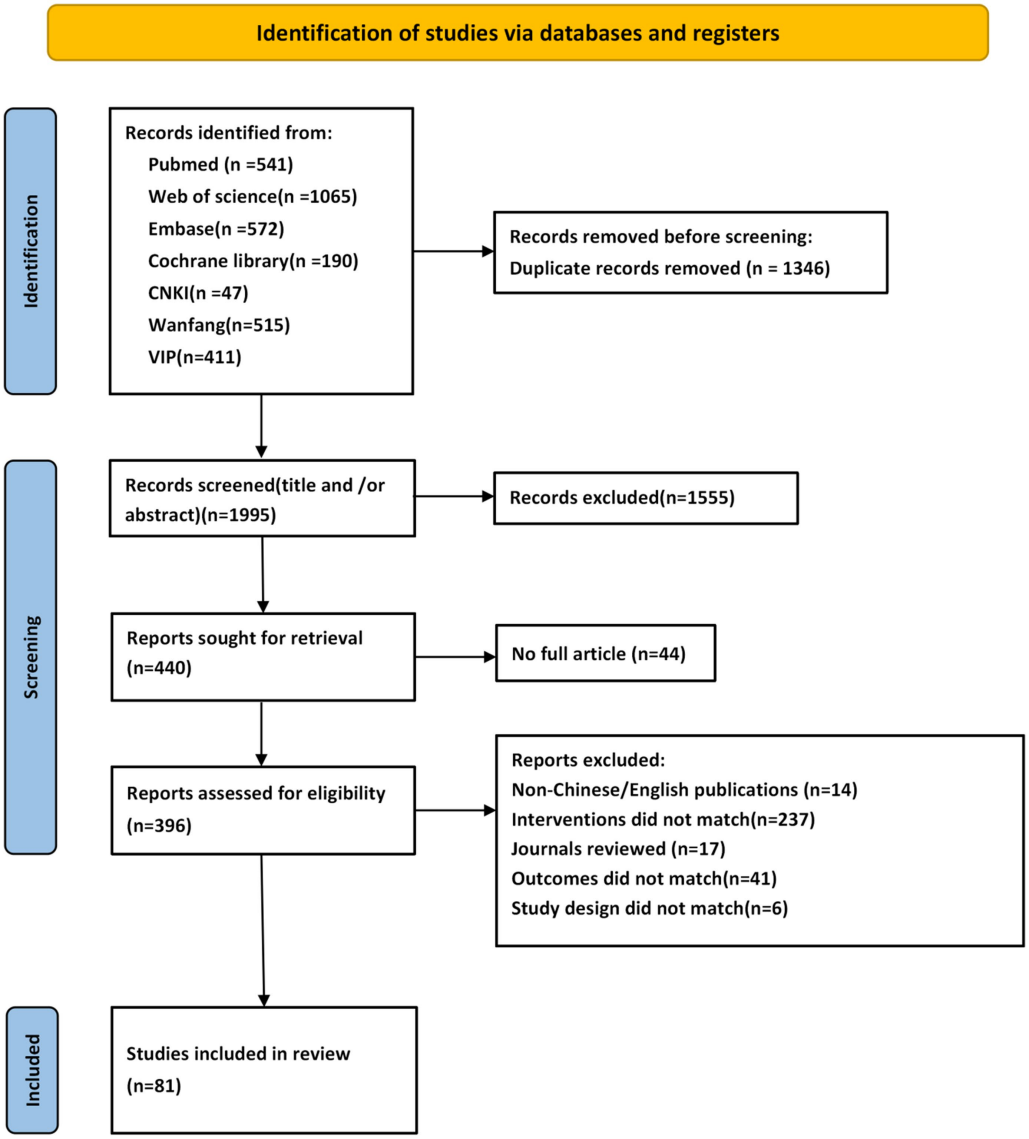
Flowchart of literature screening.

The systematic search yielded 3,341 records distributed as follows: CNKI (n = 47), Wanfang Data (n = 515), VIP (n = 411), PubMed (n = 541), Web of Science (n = 1,065), Embase (n = 572), and Cochrane Library (n = 190). EndNote X9 (2025 release) identified and eliminated 1,346 duplicate records, resulting in 81 eligible studies for final inclusion. The detailed screening process is shown in Figure 1.

### 3.2 Research characteristic

A total of 8 RCTs involving 6,147 stroke patients with lower limb dysfunction were included. Across both cohorts, 24 distinct interventions were implemented, comprising 18 modalities of electrical stimulation therapy: tDCS, repetitive transcranial magnetic stimulation (rTMS), FES, low-frequency electrical stimulation (LFES), Intermediate-Frequency Electrical (LFES), deep muscle electrical stimulation (DFES), NMES, transcutaneous acupoint electrical stimulation (TAES), TENS, transcutaneous auricular vagus nerve stimulation (taVNS), controllable current neuromuscular electrical stimulation (CCNEMS), muscle-triggered electrical stimulation (EMG), FES cycling training (FES cycling), dual-channel FES (2-ch FES), quad-channel FES (4-ch FES), multi-channel FES (multi-ch FES), Fastigial Nucleus Stimulation (FNS), and multi-joint linked wearable FES (MJL-WFES). Among the 86 included studies, 62 evaluated FMA-L scores pre- and post-treatment. Furthermore, 38 studies utilized the BBS, 26 employed the MBI pre−/post-intervention, 11 implemented the 10MWT, and 9 applied the FAC. Basic characteristics of the included literature are presented in Table 1.

**Table 1:** Characteristics of included studies.

Study ID	Grouping method	Age (C/I)	Gender (male/female)	Sample size(C/I)	Course of disease	Control group	Intervention group	Treatment Course	Outcome
Liang X 2023 (63)	A	C: 66.25 ± 12.04 I: 66.11 ± 12.16	C: 26/17 I: 28/15	43/43	C: 3.45 ± 1.16 (m) I: 3.33 ± 1.23	C: UC	I: tDCS	3 weeks	②
Song L 2021 (64)	A	C: 61.17 ± 13.56 I: 61.92 ± 11.61	C: 16/4 I: 17/3	20/20	C: 17.69 ± 2.30(d) I: 18.50 ± 1.84	C: UC	I: FES-Cycling	4 weeks	①⑤
Wei J 2024 (65)	A	C: 68.41 ± 3.46 I: 68.95 ± 3.32	C: 52/47 I: 55/43	99/99	C: 34.73 ± 6.58(d) I: 34.86 ± 6.86	C: UC	I: FES	8 weeks	①②
Hu B 2022 (66)	A	C: 71.45 ± 2.14 I: 71.73 ± 2.48	C: 23/18 I: 24/17	41/41	C: 2.29 ± 0.44(m) I: 2.17 ± 0.33	C: UC	I: LFES	4 weeks	④
Sun X 2023 (67)	B	C: 65.17 ± 4.72 I: 64.98 ± 4.63	C: 37/41 I: 48/30	78/78	C: 1.69 ± 0.71(m) I: 1.65 ± 0.78	C: UC	I: LFES	8 weeks	①②③
Hong Y 2021 (68)	A	C: 49.82 ± 7.45 I: 50.23 ± 7.68	C: 28/19 I: 26/21	47/47	C: 18.67 ± 2.35(d) I: 19.74 ± 3.06	C: UC	I: LFES	4 weeks	①②
Zhang S 2023 (69)	A	C: 68.30 ± 3.35 I: 68.70 ± 3.40	C: 26/23 I: 25/24	49/49	C: 3.04 ± 0.60(d) I: 3.19 ± 0.78	C: UC	I: LFES	4 weeks	②⑤
Dong Y 2022 (70)	A	C: 64.85 ± 11.96 I: 63.67 ± 11.39	C: 45/15 I: 42/18	60/60	C: 41.84 ± 21.74(d) I: 40.17 ± 22.65	C: UC	I: LFES	12 weeks	②
Li L 2024 (71)	A	C: 62.96 ± 4.41 I: 62.85 ± 4.37	C: 28/22 I: 30/20	50/50	C: 35.47 ± 4.52(d) I: 34.58 ± 4.96	C: UC	I: LFES	12 weeks	②
Liang S 2022 (72)	A	C: 66.78 ± 6.53 I: 66.64 ± 6.29	C: 19/20 I: 22/18	39/40	C: 36.59 ± 10.25(d) I: 36.67 ± 10.33	C: FES	I: rTMS	3 weeks	①
Zong T 2012 (73)	A	C: 62.7 ± 5.28 I: 4.5 ± 6.54	C: 24/16 I: 23/17	40/40	C: 23.6 ± 7.08(d) I: 24.5 ± 5.88	C: UC	I: IFES	4 weeks	③
Wang L 2023 (74)	A	C: 59.20 ± 7.94 I: 59.75 ± 7.58	C: 8/12 I: 9/11	20/20	C: 61.40 ± 11.21(d) I: 61.05 ± 11.45	C: UC	I: multi-ch FES	4 weeks	①④
Shi Z (75) 2023	A	C: 67.39 ± 2.20 I: 67.43 ± 2.26	C: 26/23 I: 25/24	49/49	C: 26.20 ± 2.58(d) I: 26.24 ± 2.61	C: UC	I: multi-ch FES	4 weeks	①②⑤
Liu D 2020 (15)	C	C: 53.97 ± 3.52 I: 53.72 ± 3.47	C: 9/4 I: 7/6	13/12	C: 62.27 ± 3.56(d) I: 63.43 ± 4.72	C: UC	I: FES	3 weeks	①⑤
Wang Y 2018 (76)	B	C: 64.20 ± 7.22 I: 62.50 ± 5.19	C: 6/4 I: 7/3	10/10	C: 16.56 ± 1.67 I: 16.10 ± 1.79	C: UC	I: FES-Cycling	4 weeks	①③
Ren H 2018 (77)	A	C: 68.53 ± 10.37 I1: 70.65 ± 9.98 I2: 67.32 ± 8.79 I3: 69.77 ± 7.86	C: 13/7 I1: 11/9 I2: 13/7 I3: 10/10	20/20/20/20	C: 5.31 ± 1.73(m) I1: 4.52 ± 2.21 I2: 5.16 ± 1.99 I3: 4.78 ± 2.07	C: UC	I1: EMG-FES I2: FES I3: EMG-FES + FES	8 weeks	①④
Xu C 2024 (78)	B	C: 43.32 ± 4.64 I: 44.43 ± 4.58	C: 26/26 I: 28/23	52/52	C: 1.68 ± 0.23(M) I: 1.61 ± 0.25	C: UC	I: FES	6 weeks	①
Chen D 2020 (79)	B	C: 65.510.2 I: 64.9 ± 10.5	C: 16/14 I: 18/12	20/20	C: 21.08 ± 6.18(d) I: 21.39 ± 6.18	C: UC	I: FES	2 weeks	①②④⑤
Ding B 2022 (80)	A	C: 68.2 ± 4.6 I: 69.1 ± 4.7	C: 20/18 I: 22/16	38/38	C: 39.3 ± 5.89d(d) I: 40.2 ± 5.7	C: UC	I: FES	2 weeks	①②
Ma Q 2016 (81)	A	C: 54.59 ± 9.47 I: 53.57 ± 8.04	C: 11/12 I: 10/12	22/23	C: 85.43 ± 23.89(d) I: 84.27 ± 22.42	C: UC	I: FES	8 weeks	①④
Liu Y 2021 (17)	D	C: 44.00 ± 9.58 I: 45.90 ± 8.93	C: 11/9 I: 10/10	20/20	C: 3.40 ± 1.02(m) I: 3.60 ± 1.25	C: UC I: C + FES	I: FES	4 weeks	④
Sun B 2020 (82)	A	I: 56.02 ± 8.22 C: 55.94 ± 8.38	I: 24/17 C: 22/19	41/41	I: 2.26 ± 0.27(m) C: 2.21 ± 0.39	C: UC	I: FES	NR	①②
Zheng L 2014 (83)	B	C: 53.6 ± 9.7 I: 57.6 ± 8.5	C: 10/6 I: 12/4	16/16	C: 25.5 ± 5.0(d) I: 29.5 ± 7.0	C: UC	I: FES	4 weeks	①②
Xu Y 2023 (84)	A	C: 61.00 ± 5.03 I: 60.16 ± 6.52	C: 24/18 I: 22/20	42/42	C: 25.47 ± 3.00(d) I: 26.21 ± 3.49	C: 2-ch FES	I: multi-ch FES	3 weeks	①②③
Ma Y 2011 (85)	B	53.80 ± 13.04	39/21	30/30	103.56 ± 22.48(d)	C: UC	I: TENS	4 weeks	①
Cao L 2022 (86)	A	C: 60.24 ± 6.56 I: 59.87 ± 6.61	C: 30/26 I: 32/24	56/56	C: 3.21 ± 0.84(m) I: 3.26 ± 0.80	C: UC	I: TENS	4 weeks	①③
Peng Y 2015 (18)	E	C: 68.8 ± 10.6 I: 65.4 ± 12.8	C:11/9 I:12/9	20/21	C: 1.85 ± 1.15(m) I: 2.05 ± 0.85	C: UC	I: TEAS	3 weeks	①②
Chen C 2016 (87)	B	C: 60.59 ± 10.75 I: 59.38 ± 9.59	C: 13/4 I: 14/2	17/16	C: 67.76 ± 30.87(d) I: 65.44 ± 29.25	C: UC	I: TEAS	4 weeks	①③
Wang Q 2023 (88)	A	C: 58.46 ± 8.95 I: 57.60 ± 9.41	C: 24/14 I: 26/12	38/38	C: 42.74 ± 17.28(d) I: 41.63 ± 18.03	C: UC	I: TEAS	4 weeks	①②
Wang Z 2024 (31)	A	C: 66.48 ± 10.91 I: 65.87 ± 10.79	C: 55/27 I: 59/24	82/83	C: 47.08 ± 7.36(d) I: 46.21 ± 7.22	C: UC	I: TEAS+tDCS	4 weeks	①④⑤
Gong X 2024 (89)	A	C: 56.53 ± 11.02 I: 60.42 ± 13.21	C: 10/5 I: 11/4	15/15	C: 2.12 ± 2.78(m) I: 2.34 ± 2.2	C: UC	I: tDCS	2 weeks	②
Zhang Q 2025 (90)	A	C: 63.23 ± 6.70 I: 61.22 ± 8.65	C: 23/12 I: 28/8	35/36	C: 3.91 ± 1.01 I: 4.00 ± 0.76	C: UC	I: DEMS	4 weeks	①
Qiu Z 2022 (91)	A	C: 61.45 ± 3.35 I: 61.33 ± 3.27	C: 15/26 I: 14/27	41/41	C: 20.45 ± 3.15(d) I: 20.36 ± 3.02	C: UC	I: NEMS	12 weeks	②③
Yang M 2023 (92)	A	C: 61.34 ± 3.55 I: 61.72 ± 2.15	C: 39/36 I: 38/37	75/75	C: 4.51 ± 0.32(m) I: 4.59 ± 0.24	C: UC	I: NEMS	4 weeks	①②
Wen X 2021 (93)	B	C: 66.1 ± 3.7 I: 65.3 ± 3.2	C: 26/18 I: 24/20	44/44	C: 4.19 ± 0.40(w) I: 4.25 ± 0.36	C: UC	I: NEMS	4 weeks	①
Yan X 2023 (94)	B	C: 63.21 ± 3.11 I: 63.23 ± 3.14	C: 18/22 I: 21/19	40/40	C: 4.57 ± 1.03(w) I: 4.59 ± 1.01	C: UC	I: NEMS	10 weeks	③
Zhang G 2023 (95)	A	C: 60.37 ± 5.47 I: 60.33 ± 5.54	C: 24/26 I: 25/25	50/50	C: 2.03 ± 0.44(m) I: 2.13 ± 0.38	C: UC	I: IFES	8 weeks	②
Wang L 2024 (25)	F	I1: 64.93 ± 10.54 I2: 61.65 ± 9.33 C: 61.00 ± 9.67	I1: 11/16 I2: 8/17\u00B0C: 6/14	27/25/20	I1: 12.37 ± 6.15 (d) I2: 16.42 ± 4.71 C: 15.86 ± 6.59	C: UC	I1: rTMS I2: Placebo	6 weeks	①②③
Zhang X. H. 2021 (96)	A	C: 57.31 ± 10.53 I: 55.76 ± 11.78	C: 16/17 I: 13/20	33/33	C: 42.45 ± 4.75(d) I: 43.55 ± 5.66	C: UC	I: FES-Cycling	8 weeks	①③
Bilek, F 2020 (28)	B	C: 62.6 ± 2.2 I: 51.3 ± 3.7	C: 16/14 I: 13/17	30/30	3 months	C: UC	I: NEMS	6 weeks	⑤
Shuji, M 2023 (22)	G	C: 64.3 ± 11.8 I: 63.5 ± 10.5	C: 70/22 I: 68/24	92/92	C: 63.7 ± 30.4(d) I: 59.5 ± 32.6	C: UC	I: FES	8 weeks	①④
Zhang X 2021 (32)	B	C: 58.18 ± 11.70 I: 56.11 ± 12.0	C: 38/23 I: 43/18	61/61	C: 43.45 ± 5.66(d) I: 42.45 ± 4.75	C: FES	I: tDCS	8 weeks	①③⑤
Ying Shen 2022 (24)	F	C: 66.09 ± 6.38 I: 62.86 ± 12.96	C: 15/6 I: 18/3	21/21	C: 73.45 ± 33.15 I: 84.00 ± 39.60(d)	C: NEMS	I: CCNEMS	3 weeks	①③
Tan Z 2014 (30)	A	C: 67.0 ± 9.0 I1: 63.4 ± 10.6 I2: 64.6 ± 8.3	C: 8/9 I1: 8/8 I2: 8/6	17/16/15	C: 41.5 ± 20.4(d) I1: 41.3 ± 29.4 I2: 41.6 ± 22.1	C: UC	I1: 4-ch FES I2: 2-ch FES	3 weeks	①②③⑤
Jung K. S 2020 (16)	C	C: 52.7 ± 11.5 I: 53.1 ± 7.9	C: 12/8 I: 14/6	20/20	C: 7.0 ± 2.6(m) I: 6.8 ± 2.5	C: Placebo	I: TENS	6 weeks	④
Hsu S. P 2023 (97)	B	C: 59.2 ± 11.8 I: 59.1 ± 11.4	C: 6/8 I: 9/4	13/14	C: 21.1 ± 5.3(d) I: 20.7 ± 3.5	C: UC	I: tDCS	2 weeks	①
Litong Wang 2024 (98)	A	C: 61.94 ± 3.28 I1: 61.54 ± 5.78 I2: 60.82 ± 6.19 I3: 63.13 ± 5.75	C: 17/23 I1: 22/21 I2: 21/23 I3: 18/24	40/43/44/42	C: 18.89 ± 5.17(d) I1: 18.98 ± 4.56 I2: 619.24 ± 5.83 I3: 20.05 ± 4.28	C: UC	I1: taVNS+tDCS I2: taVNS I3: tDCS	4 weeks	①②③
Dujovic S. D. 2017 (99)	B	65	C: 7/1 I: 3/5	8/8	6 (m)	C: UC	I: FES	4 weeks	①②③
Zheng X 2018 (100)	B	C: 59 ± 9 I1: 59 ± 11 I2: 60 ± 9	C: 9/6 I1: 9/9 I2: 9/6	15/18/15	C: 20 ± 12(d) I1: 20 ± 11 I2: 21 ± 13	C: UC	I1: 4-ch FES I2: 2-ch FES	3 weeks	①②③
Yang T 2018 (101)	F	C: 60.60 ± 8.33 I: 63.07 ± 4.46	C: /6 I: /7	15/15	C: 7.40 ± 14.51(d) I: 9.53 ± 15.90	C: NEMS	I: FES	4 weeks	①④
Xu J 2015 (102)	B	C: 63.1 ± 10.1 I: 65.6 ± 12.7	C: 4/16 I: 7/13	40/40	2.5-8(d)	C: UC	I: NEMS	2 weeks	①
Li J 2023 (23)	I	C: 65.19 ± 7.15 I: 65.26 ± 7.04	C: 25/18 I: 22/20	43/42	C: 5.28 ± 0.67(d) I: 5.36 ± 0.67	C: UC	I: LFES	12 weeks	①②
Chen J 2006 (103)	B	C: 61.5 ± 3.5 I: 62.0 ± 3.2	C: 17/8 I: 16/9	25/25	1 ~ 4 days	C: UC	I: LFES	20 days	①
Yan G 2009 (29)	B	C: 54.8 ± 6.3 I: 55.2 ± 6.8	C: 17/13 I: 19/12	30/31	C: 4.7 ± 0.5(d) I: 4.6 ± 0.5	C: UC	I: LFES	2 weeks	①③
Zhou J 2003 (104)	B	C: 62.38 ± 11.5 I: 59.35 ± 8.75	C: 22/18 I: 21/19	40/40	≤72 h	C: UC	I: FNS	10 days	①
Liu J 2024 (27)	S	C: 50.73 ± 11.25 I: 49.13 ± 10.79	C: 9/6 I: 11/14	15/15	C: 6.60 ± 3.42(m) I: 6.67 ± 3.64	C: UC	I: MJL-WFES	4 weeks	①③
Xu D 2019 (105)	B	C: 57.49 ± 9.9 I: 54.09 ± 14.73	C: 20/15 I: 25/10	35/35	C: 2.91 ± 2.32(m) I: 2.49 ± 1.67	C: UC	I: multi-ch FES	4 weeks	①
Jin G 2017 (26)	H	C: 59.4 ± 6.2 I: 59.6 ± 6.1	C: 18/16 I: 20/14	34/34	<1 week	C: 2-ch FES	I: multi-ch FES	3 weeks	①②
Chen D 2013 (106)	B	C: 56.0 ± 8.8 I: 50.5 ± 10.7	C: 6/2 I: 7/3	8/10	C: 15.9 ± 6.0(d) I: 17.0 ± 8.2	C: UC I: FES	I: FES	3 weeks	①②
Liu J 2021 (107)	A	C: 55.10 ± 14.01 I: 58.43 ± 9.52	C: 8/7 I: 9/6	15/15	C: 6.47 ± 1.83(m) I: 6.73 ± 1.81	C: UC	I: multi-ch FES	5 weeks	①③
Li G 2019 (108)	A	C: 58.40 ± 7.02 I: 58.53 ± 7.13	C: 16/14 I: 17/13	30/30	C:12.67 ± 7.56(d) I:13.07 ± 8.13	C: UC	I: FES	2 weeks	①②
Zhan S 2021 (109)	A	C: 63.6 ± 5.3 I: 65.1 ± 4.8	C: 43/30 I: 47/26	73/73	C: 0.6 ± 0.3(m) I: 0.7 ± 0.2	C: UC	I: FES	3 weeks	②
You G 2007 (20)	E	C: 64.1 ± 9.7 I: 60.8 ± 10.8	C: 10/8 I: 11/8	18/19	C: 22.7 ± 16.6(d) I: 25.9 ± 21.3	C: UC	I: 2-ch FES	3 weeks	①②
You G 2013 (19)	E	C: 64.2 ± 8.8 I1: 62.7 ± 10.9 I2: 61.6 ± 9.7	C: 15/7 I1: 13/10 I2: 14/9	22/23/23	C: 25.2 ± 19.4(d) I1: 24.6 ± 20.2 I2: 23.7 ± 16.9	C: UC	I1: 2-ch FES I2: Placebo	3 weeks	①②
Cai C 2021 (110)	B	C: 56.43 ± 13.05 I: 57.20 ± 13.48	C: 74/26 I: 77/23	100/100	NR	C: UC	I: FES	8 weeks	①②④
Yang L 2017 (111)	A	C: 58.6 ± 13.8 I: 59.1 ± 13.3	C: 18/12 I: 18/12	30/30	C: 17.4 ± 5.8(d) I: 17.7 ± 6.3	C: UC	I: EMG-FES	8 weeks	①②
Zhao J 2023 (112)	A	C: 54.98 ± 4.32 I: 54.37 ± 4.54	C: 12/8 I: 10/10	20/20	C: 13.65 ± 2.31(d) I: 13.87 ± 2.42	C: UC	I: tDCS	4 weeks	①
Guo T 2015 (113)	B	C: 59.59 ± 10.02 I: 58.33 ± 9.26	C: 20/20 I: 19/11	30/30	NR	C: UC	I: tDCS	4 weeks	③
Geng J 2021 (114)	A	C: 57.37 ± 13.16 I: 60.50 ± 13.21	C: 19/12 I: 18/13	31/31	C: 12.11 ± 2.08(d) I: 11.84 ± 1.8	C: FES-cycling	I: tDCS	4 weeks	①③
Chen H 2021 (21)	E	C: 50.1 ± 11.5 I1: 44.4 ± 12.8 I2: 48.4 ± 13.2	C: 14/4 I1: 14/5 I2: 13/6	18/19/19	3-6(m)	C: tDCS	I1: FES I2: tDCS+FES	12 days	①②③
Cheng A 2005 (115)	A	58 ± 5	38/22	30/30	NR	C: UC	I: TENS	4 weeks	①
Zhang H 2013 (116)	B	C: 65.23 ± 9.21 I: 64.89 ± 7.73	C: 26/24 I: 25/25	50/50	C:26.35 ± 15.23(d) I:25.62 ± 12.67	C: UC	I: TEAS	2 weeks	③
Chen X 2025 (117)	A	C: 63.33 ± 1.36 I: 63.36 ± 1.39	C: 21/13 I: 21/14	34/35	NR	C: UC 1次/d	I: LFES	8 weeks	②
Wang P 2023 (118)	A	C: 45.98 ± 6.25 I: 46.86 ± 6.87	C: 14/15 I: 16/13	29/29	C: 12.95 ± 3.52(d) I: 13.98 ± 3.67	C: UC	I: DEMS	8 weeks	②
Yang Y 2015 (119)	A	65.8 ± 9.2	126/34	80/80	NR	C: UC	I: NEMS	12 weeks	③
Yang Y 2016 (120)	A	C: 63.62 ± 12.81 I: 62.73 ± 12.47	C: 22/18 I: 22/18	40/40	NR	C: UC	I: NEMS	8 weeks	①
Li X 2021 (121)	B	C: 56.26 ± 9.36 I: 59.21 ± 9.25	C: 32/28 I: 34/26	60/60	<3 months	C: UC	I: NMES	4 weeks	①②
Chen Y 2021 (122)	B	C: 68.31 ± 7.80 I1: 63.37 ± 10.99 I2: 62.35 ± 10.80	C: 10/6 I1: 8/8 I2: 6/11	16/16/16	C: 6.58 ± 2.16(m) I1: 6.66 ± 2.24 I2: 5.81 ± 2.09	C: Placeo	I1: 2-ch FES I2: 4-ch FES	2 weeks	①②
Deng W 2023 (123)	A	C: 60.56 ± 1.15 I: 60.62 ± 1.21	C: 25/15 I: 23/17	40/40	NR	C: CRT	I: IFES	4 weeks	①
Sun L 2014 (124)	A	C: 63.95 I: 65.95	C: 6/14 I: 14/6	20/20	6.8 weeks	C: UC	I: EMG-FES	8 weeks	①
Yu J 2010 (125)	B	C: 52.0 ± 6.8 I1: 51.4 ± 7.2 I2: 51.8 ± 6.2	C: 11/9 I1: 8/12 I2: 11/9	20/20/20	C: 21.6 ± 0.93(d) I1: 22.8 ± 8.8 I2: 20.2 ± 10.1	C: UC	I1: FES I2: EMG-FES	8 weeks	①

A: Random number table; B: Random; C: Treatment modality; D: Order of admission; E: Minimize; F: Sealed envelope; G: eClinical Base; H: Lottery method; I: Mendelian Randomization; S: SPSS 25.0; UC: Usual Care. The meaning of the number represented in the Outcome details: ①FMA-L score, ② BBS score, ③ MBI score, ④ 10MWT, ⑤AC score.

### 3.3 Risk of bias of included studies

**Figure 2:**
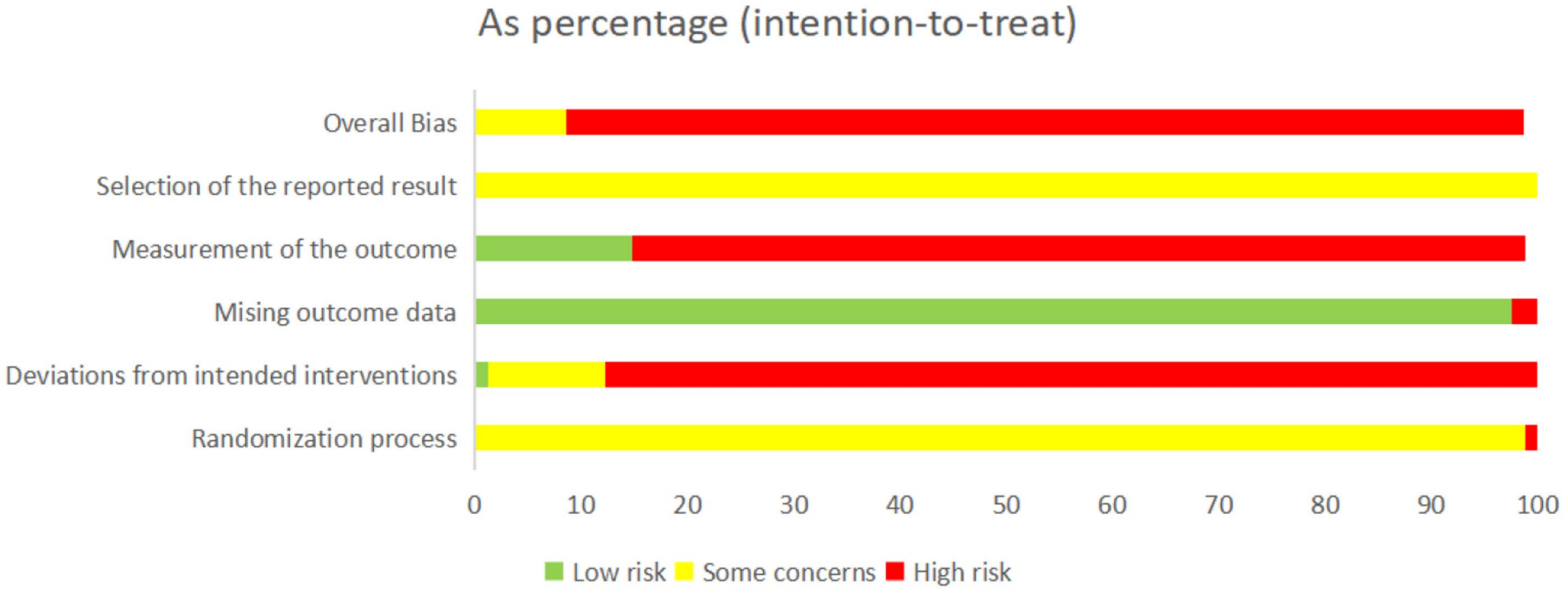
Risk of bias graph.

Regarding the randomization process, 54 studies (64.7%) clearly reported the methods used to generate random allocation sequences. Among them, 41 trials employed random number tables, two allocated participants (15, 16) utilized treatment-based allocation; 1 study (17) used admission chronology; 4 studies (18–21) mplemented Minimization methodology; 1 study (22) applied eClinical Base platform; 1 study (23) adopted Mendelian randomization; 2 studies (24, 25) applied sealed envelope concealment; 1 study (26) utilized lottery allocation; and 1 study (27) employed SPSS 25.0 software. The remaining 27 studies (35.3%) mentioned the use of “randomization” without specifying the method. To evaluate baseline comparability after randomization, we systematically reviewed the baseline data for age, sex distribution, and primary outcome measures as presented in Table 1. Most studies demonstrated balanced baseline characteristics between intervention groups (*p* > 0.05) and were therefore rated as having a low risk of bias. A small number of trials—for example, the study by Bilek (28), where age distributions differed significantly between groups—were judged to have a high risk of bias. With respect to outcome completeness, three studies exhibited incomplete data due to participant attrition, indicating a potential risk of attrition bias. No evidence of selective reporting or other identifiable sources of bias was detected. Taken together, the methodological quality of the included studies was generally modest. The detailed distribution of risk-of-bias assessments is presented in Figure 2 and Table 2.

**Table 2:** Risk of bias (RoB 2.0) summary for included trials.

Study ID	Randomization process	Deviations from intended interventions	Missing outcome data	Measurement of the outcome	Selection of the reported result	Overall
Liang X 2023 (63)	Some concerns	High risk	Low risk	High risk	Some Concerns	High risk
Song L 2021 (64)	Some concerns	High risk	Low risk	High risk	Some Concerns	High risk
Wei J 2024 (65)	Some concerns	High risk	Low risk	High risk	Some Concerns	High risk
Hu B 2022 (66)	Some concerns	High risk	Low risk	High risk	Some Concerns	High risk
Sun X 2023 (67)	Some concerns	High risk	Low risk	High risk	Some Concerns	High risk
Hong Y 2021 (68)	Some concerns	High risk	Low risk	High risk	Some Concerns	High risk
Zhang S 2023 (69)	Some concerns	High risk	Low risk	High risk	Some Concerns	High risk
Dong Y 2022 (70)	Some concerns	High risk	Low risk	High risk	Some Concerns	High risk
Li L 2024 (71)	Some concerns	High risk	Low risk	High risk	Some Concerns	High risk
Liang S 2022 (72)	Some concerns	High risk	Low risk	High risk	Some Concerns	High risk
Zong T 2012 (73)	Some concerns	High risk	Low risk	High risk	Some Concerns	High risk
Wang L 2023 (74)	Some concerns	High risk	Low risk	High risk	Some Concerns	High risk
Shi Z 2023 (75)	Some concerns	High risk	Low risk	High risk	Some Concerns	High risk
Liu D 2020 (15)	Some concerns	High risk	Low risk	High risk	Some Concerns	High risk
Wang Y 2018 (76)	Some concerns	High risk	Low risk	High risk	Some Concerns	High risk
Ren H 2018 (77)	Some concerns	High risk	Low risk	High risk	Some Concerns	High risk
Xu C 2024 (78)	Some concerns	High risk	Low risk	High risk	Some Concerns	High risk
Chen D 2020 (79)	Some concerns	High risk	Low risk	High risk	Some Concerns	High risk
Ding B 2022 (80)	Some concerns	High risk	Low risk	High risk	Some Concerns	High risk
Ma Q 2016 (81)	Some concerns	High risk	Low risk	High risk	Some Concerns	High risk
Liu Y 2021 (17)	Some concerns	High risk	Low risk	High risk	Some Concerns	High risk
Sun B 2020 (82)	Some concerns	High risk	Low risk	High risk	Some Concerns	High risk
Zheng L 2014 (83)	Some concerns	High risk	Low risk	High risk	Some Concerns	High risk
Xu Y 2023 (84)	Some concerns	High risk	Low risk	High risk	Some Concerns	High risk
Ma Y 2011 (85)	Some concerns	High risk	Low risk	High risk	Some Concerns	High risk
Cao L 2022 (86)	Some concerns	High risk	Low risk	High risk	Some Concerns	High risk
Peng Y 2015 (18)	Some concerns	High risk	Low risk	High risk	Some Concerns	High risk
Chen C 2016 (87)	Some concerns	High risk	Low risk	High risk	Some Concerns	High risk
Wang Q 2023 (88)	Some concerns	High risk	Low risk	High risk	Some Concerns	High risk
Wang Z 2024 (31)	Some concerns	High risk	Low risk	High risk	Some Concerns	High risk
Gong X 2024 (89)	Some concerns	High risk	Low risk	High risk	Some Concerns	High risk
Zhang Q 2025 (90)	Some concerns	High risk	Low risk	High risk	Some Concerns	High risk
Qiu Z 2022 (91)	Some concerns	High risk	Low risk	High risk	Some Concerns	High risk
Yang M 2023 (92)	Some concerns	High risk	Low risk	High risk	Some Concerns	High risk
Wen X 2021 (93)	Some concerns	High risk	Low risk	High risk	Some Concerns	High risk
Yan X 2023 (94)	Some concerns	High risk	Low risk	High risk	Some Concerns	High risk
Zhang G 2023 (95)	Some concerns	Some concerns	Low risk	Low risk	Some Concerns	Some Concerns
Wang L 2024 (25)	Some concerns	High risk	Low risk	High risk	Some Concerns	High risk
Zhang X. H 2021 (96)	Some concerns	Some concerns	Low risk	Low risk	Some Concerns	Some Concerns
Bilek, F 2020 (28)	High risk	Some concerns	Low risk	Low risk	Some Concerns	High risk
Shuji, M 2023 (22)	Some concerns	High risk	Low risk	High risk	Some Concerns	High risk
Zhang X 2021 (32)	Some concerns	Some concerns	Low risk	Low risk	Some Concerns	Some Concerns
Ying Shen 2022 (24)	Some concerns	High risk	Low risk	High risk	Some Concerns	High risk
Tan Z 2014 (30)	Some concerns	Some concerns	Low risk	Low risk	Some Concerns	Some Concerns
Jung K. S 2020 (16)	Some concerns	Some concerns	Low risk	Low risk	Some Concerns	Some Concerns
Hsu S. P 2023 (97)	Some concerns	Some concerns	Low risk	Low risk	Some Concerns	Some Concerns
Litong W 2024 (98)	Some concerns	High risk	Low risk	High risk	Some Concerns	High risk
Dujovic 2017 (99)	Some concerns	High risk	High risk	High risk	Some Concerns	High risk
Zheng X 2018 (100)	Some concerns	Some concerns	Low risk	Low risk	Some Concerns	Some Concerns
Yang T 2018 (101)	Some concerns	High risk	Low risk	High risk	Some Concerns	High risk
Xu J 2015 (102)	Some concerns	High risk	Low risk	High risk	Some Concerns	High risk
Li J 2023 (23)	Some concerns	High risk	Low risk	High risk	Some Concerns	High risk
Chen J 2006 (103)	Some concerns	High risk	Low risk	High risk	Some Concerns	High risk
Yan G 2009 (29)	Some concerns	Low risk	Low risk	High risk	Some Concerns	High risk
Zhou J 2003 (104)	Some concerns	High risk	High risk	High risk	Some Concerns	High risk
Liu J 2024 (27)	Some concerns	High risk	Low risk	High risk	Some Concerns	High risk
Xu D 2019 (105)	Some concerns	High risk	Low risk	High risk	Some Concerns	High risk
Jin G 2017 (26)	Some concerns	High risk	Low risk	High risk	Some Concerns	High risk
Chen D 2013 (106)	Some concerns	High risk	High risk	High risk	Some Concerns	High risk
Liu J 2021 (107)	Some concerns	High risk	Low risk	High risk	Some Concerns	High risk
Li G 2019 (108)	Some concerns	High risk	Low risk	High risk	Some Concerns	High risk
Zhan S 2021 (109)	Some concerns	High risk	Low risk	High risk	Some Concerns	High risk
You G 2007 (20)	Some concerns	High risk	Low risk	High risk	Some Concerns	High risk
You G 2013 (19)	Some concerns	High risk	Low risk	High risk	Some Concerns	High risk
Cai C 2021 (110)	Some concerns	High risk	Low risk	High risk	Some Concerns	High risk
Yang L 2017 (111)	Some concerns	High risk	Low risk	High risk	Some Concerns	High risk
Zhao J 2023 (112)	Some concerns	High risk	Low risk	High risk	Some Concerns	High risk
Guo T 2015 (113)	Some concerns	High risk	Low risk	High risk	Some Concerns	High risk
Geng J 2021 (114)	Some concerns	High risk	Low risk	High risk	Some Concerns	High risk
Chen H 2021 (21)	Some concerns	High risk	Low risk	High risk	Some Concerns	High risk
Cheng A 2005 (115)	Some concerns	High risk	Low risk	High risk	Some Concerns	High risk
Zhang H 2013 (116)	Some concerns	High risk	Low risk	High risk	Some Concerns	High risk
Chen X 2025 (117)	Some concerns	High risk	Low risk	High risk	Some Concerns	High risk
Wang P 2023 (118)	Some concerns	High risk	Low risk	High risk	Some Concerns	High risk
Yang Y 2015 (119)	Some concerns	High risk	Low risk	High risk	Some Concerns	High risk
Yang Y 2016 (120)	Some concerns	High risk	Low risk	High risk	Some Concerns	High risk
Li X 2021 (121)	Some concerns	High risk	Low risk	High risk	Some Concerns	High risk
Chen Y 2021 (122)	Some concerns	High risk	Low risk	High risk	Some Concerns	High risk
Deng W 2023 (123)	Some concerns	High risk	Low risk	High risk	Some Concerns	High risk
Sun L 2014 (124)	Some concerns	High risk	Low risk	High risk	Some Concerns	High risk
Yu J 2010 (125)	Some concerns	High risk	Low risk	High risk	Some Concerns	High risk

### 3.4 Pairwise meta-analysis

We performed pairwise meta-analyses for all interventions, yielding 5 outcomes. Forest plots and heterogeneity analyses for the pairwise meta-analysis results are presented in Supplementary material 3. Heterogeneity analysis revealed high heterogeneity across most electrical stimulation protocols, except for the BBS score outcome (*I^2^* = 0%). Given the potential high heterogeneity arising from varying treatment durations, we conducted subgroup analyses based on treatment duration. Detailed results are shown in Supplementary material 3. After subgroup analysis, significant heterogeneity persisted in the FMA-L score outcome for both the ≤6-week and >6-week disease duration subgroups (original *I^2^* = 94.09 and 89.59%, respectively). Heterogeneity predominantly originated from studies exhibiting extreme effect sizes (e.g., Yanguoping (29)), with the underlying mechanism being increased efficacy dispersion attributable to inter-study variations in electrical stimulation parameters (intensity/frequency). Sensitivity analysis following the exclusion of these effect size outliers significantly attenuated heterogeneity, thereby validating the therapeutic advantage of electrical stimulation for enhancing lower limb motor function in stroke patients and furnishing high-grade evidence for individualized rehabilitation protocols. In the MBI analysis, significant baseline heterogeneity was observed in both ≤6-week and >6-week subgroups (*I^2^* = 94.58% each). Heterogeneity was mainly driven by extreme effect sizes (e.g., 34.06 points in Yanguoping (29) vs. -2.10 points in Zhimei Tan (30)), attributable to efficacy dispersion caused by parameter variations. Post-exclusion sensitivity analysis substantially reduced heterogeneity (overall I^2^ decreased from 65.62 to 18.18%), confirming electrical stimulation’s benefits for improving activities of daily living. Analysis of the 10MWT revealed substantial heterogeneity in both the ≤4-week and >4-week disease duration subgroups. This was principally caused by distortion of the pooled effect size due to disproportionate weighting from an ultra-large sample study (Wangzhida (31), constituting 45% of subgroup data). Exclusion of this study yielded significant heterogeneity reduction. This revealed electrical stimulation’s significantly superior efficacy for improving walking function in acute versus chronic phases. For the FAC analysis, high heterogeneity existed in the ≤4-week subgroup (*I^2^* = 84.80%), primarily driven by the extreme effect size in Xiaohua Zhang (32) (1.41 [1.16, 1.66]), where tDCS efficacy significantly surpassed other interventions (validated by Surface Under the Cumulative Ranking Analysis (SUCRA)). The >4-week subgroup showed minimal heterogeneity (*I^2^* = 0.00%), indicating stable efficacy in chronic stages. After excluding high-heterogeneity studies, short-term treatment group heterogeneity decreased significantly (*I^2^* = 0.00%), confirming tDCS’s pivotal role in improving acute-phase walking function.

### 3.5 Network meta-analysis

**Figure 3:**
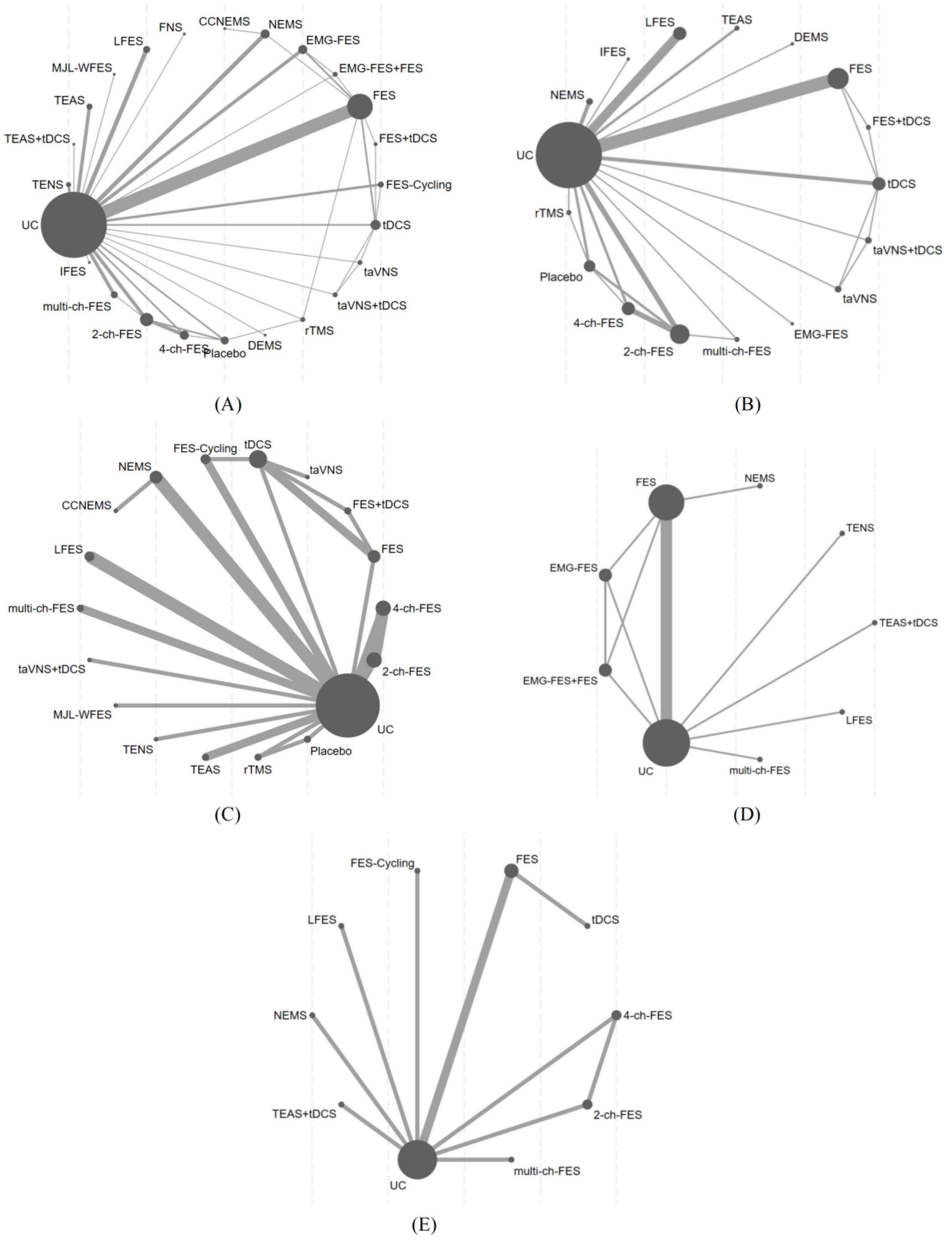
Evidence relationship diagram of different interventions in the included studies. **(A)** FMA-L score; **(B)** BBS score; **(C)** MBI score; **(D)** 10MWT; **(E)** FAC score.

**Figure 4:**
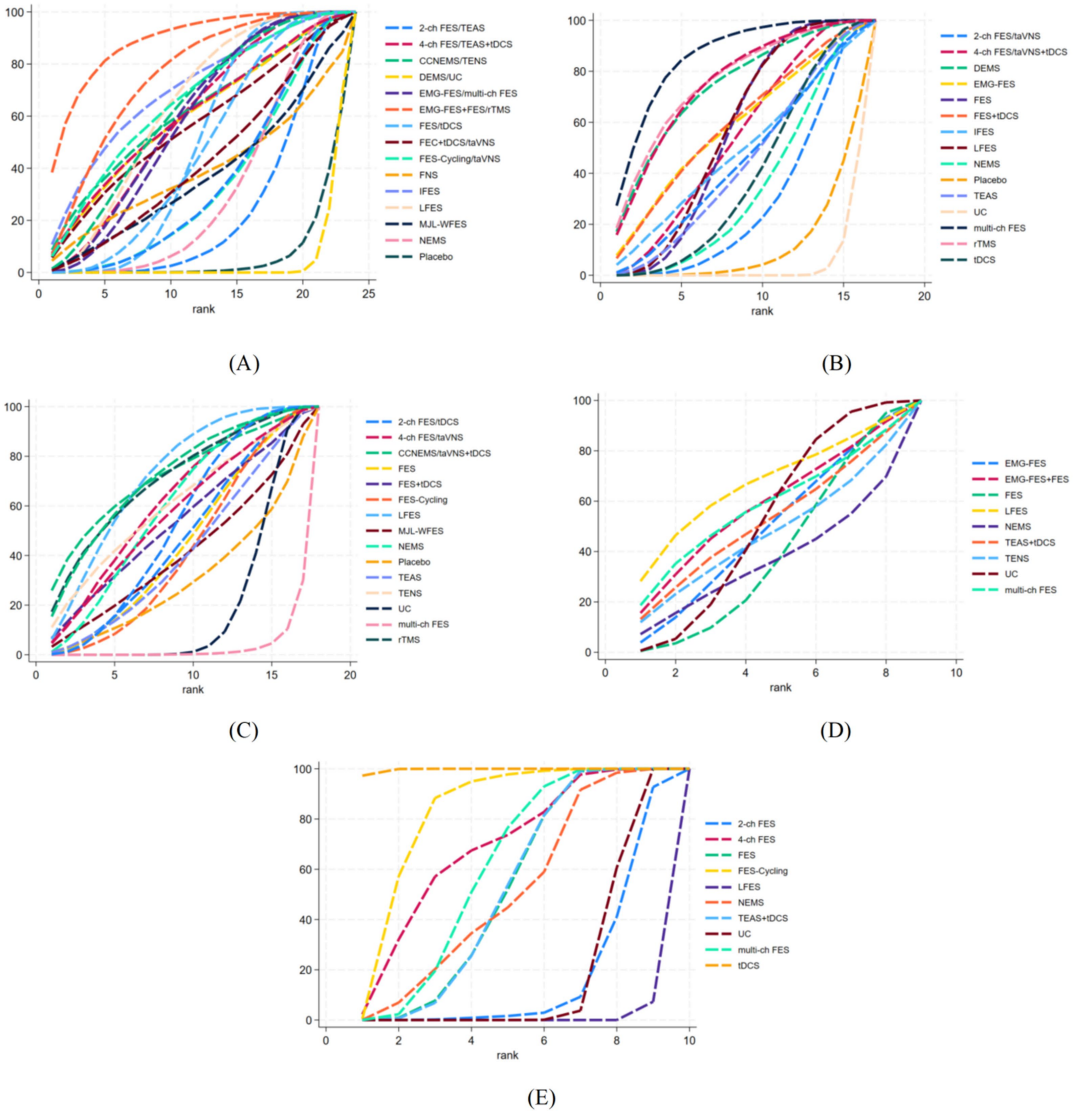
Cumulative probability plot of the therapeutic effects of different interventions in the included studies. **(A)** FMA-L score; **(B)** BBS score; **(C)** MBI score; **(D)** 10MWT; **(E)** FAC score.

The network structure of all outcomes is shown in Figure 3. We tested the convergence of all outcome models. The trace and density plots in Supplementary material 4 reveal extensive chain overlap, precluding visual discrimination of individual chain iterations. All curves approximate a normal distribution with stable bandwidth values. Brooks-Gelman-Rubin diagnostics in Supplementary material 5 demonstrate convergence: reduction factor medians and 97.5% quantiles approach 1, with all PSRF values at unity. Thus, all outcome models demonstrate excellent convergence. SUCRA values were computed for five outcomes under various interventions, with treatment rankings derived from the area under the cumulative probability curve (Figure 4).

#### 3.5.1 FMA-L

A total of 62 studies involving 4,520 subjects reported FMA-L outcomes. Interventions included: 4 FES-Cycling, 18 FES, 5 LFES, 2 rTMS, 5 multi-ch FES, 4 EMG-FES, 1 EMG-FES + FES, 4 4-ch FES, 7 2-ch FES, 3 TENS, 4 TEAS, 1 TEAS+tDCS, 1 DEMS, 7 NEMS, 5 tDCS, 1 CCNEMS, 1 MLJ-WFES, 1 taVNS+tDCS, 1 taVNS, 1 FNS, and 1 IFES. The EMG-FES + FES intervention demonstrated maximal efficacy in augmenting the area under the curve for BBS scores (SUCRA: 89.0%), contrasting with minimal efficacy observed for usual care (SUCRA: 4.3%). The SUCRA-derived hierarchy of interventions for FMA-L score enhancement was: EMG-FES + FES (89.0%) > rTMS (75.9%) > IFES (70.3%) > taVNS+tDCS (66.2%) > LFES (64.4%) > TENS (63.0%) > CCNEMS (61.7%) > TEAS+tDCS (60.8%) > EMG-FES (60.5%) > DEMS (60.1%) > 4-ch FES (59.7%) > multi-ch FES (58.4%) > FES + tDCS (56.6%) > FES (49.7%) > tDCS (47.8%) > taVNS (42.8%) > FNS (39.5%) > MLJ-WFES (37.6%) > TEAS (34.9%) > FES-Cycling (34.1%) > NEMS (32.0%) > 2-ch FES (24.0%) > Placebo (6.7%) > UC (4.3%).

#### 3.5.2 BBS

A total of 38 studies involving 3,208 subjects reported 10MWT outcomes. Interventions included: 7 tDCS, 10 FES, 7 LFES, 2 multi-ch FES, 4 4-ch FES, 7 2-ch FES, 2 TEAS, 3 NEMS, 1 IFES, 1 rTMS, 1 EMG-FES, 1 taVNS+tDCS, 1 taVNS, 1 DEMS, and 1 FES + tDCS. Multi-channel functional electrical stimulation (multi-ch FES) exhibited maximal efficacy in enhancing the area under the curve for Berg Balance Scale scores (SUCRA: 85.6%), contrasting with minimal therapeutic effect from usual care (SUCRA: 4.2%). The therapeutic hierarchy for Berg Balance Scale enhancement, derived from SUCRA metrics, was: multi-ch FES (85.6%) > rTMS (76.0%) > taVNS+tDCS (75.2%) > DEMS (73.9%) > FES + tDCS (59.2%) > EMG-FES (58.4%) > LFES (57.0%) > FES (55.7%) > 4-ch FES (53.9%) > IFES (48.6%) > taVNS (45.0%) > TEAS (44.0%) > tDCS (38.7%) > NEMS (34.4%) > 2-ch FES (28.5%) > Placebo (11.6%) > UC (4.2%).

#### 3.5.3 MBI

A total of 26 studies involving 1,886 subjects reported 10MWT outcomes. Interventions included: 3 LFES, 2 multi-ch FES, 3 FES-Cycling, 3 2-ch FES, 3 4-ch FES, 3 TENS (2 + 1 studies), 2 TEAS, 4 NEMS, 3 FES, 1 rTMS, 5 tDCS, 1 CCNEMS, 1 taVNS+tDCS, 1 taVNS, and 1 MJL-WFES. CCNEMS exhibited maximal efficacy in augmenting the area under the curve for Modified Barthel Index scores (SUCRA: 71.9%), contrasting with minimal therapeutic benefit from usual care (SUCRA: 3.0%). The therapeutic hierarchy for Modified Barthel Index enhancement, per SUCRA metrics, was: CCNEMS (71.9%) = LFES (71.9%) > taVNS+tDCS (71.7%) > rTMS (70.6%) > 4-ch FES (62.5%) > TENS (60.5%) > NEMS (59.7%) > taVNS (56.9%) > FES + tDCS (53.3%) > tDCS (52.2%) > 2-ch FES (46.3%) > FES (44.8%) > FES-Cycling (41.8%) > TEAS (41.5%) > MJL-WFES (41.0%) > Placebo (19.7%) > UC (3.0%).

#### 3.5.4 10MWT

A total of 11 studies involving 946 subjects reported 10-meter walk test (10MWT) outcomes. Interventions included: 1 LFES, 6 FES, 1 EMG-FES + FES, 1 EMG-FES, 1 TEAS+tDCS, 1 NEMS, 1 TENS, and 1 multi-ch FES. Low-frequency electrical stimulation (LFES) exhibited maximal efficacy in enhancing the area under the curve for 10-meter walk test performance (SUCRA: 66.2%), contrasting with minimal therapeutic benefit from neuroelectrical muscle stimulation (NEMS, SUCRA: 35.6%). The therapeutic hierarchy for 10-meter walk test enhancement, per SUCRA metrics, was: LFES (66.2%) > EMG-FES + FES (57.2%) > multi-ch FES (56.9%) > TEAS+tDCS (50.9%) > usual care (UC, 51.2%) > EMG-FES (47.9%) > TENS (46.0%) > FES (38.2%) > NEMS (35.6%).

#### 3.5.5 FAC

A total of 9 studies involving 715 subjects reported 10-meter walk test (10MWT) outcomes. Interventions included: 3 FES, 1 tDCS, 1 FES-Cycling, 1 4-ch FES, 1 2-ch FES, 1 TEAS+tDCS, 1 NEMS, 1 multi-ch FES, and 1 LFES. Transcranial direct current stimulation (tDCS) exhibited maximal efficacy in enhancing the area under the curve for Functional Ambulation Category measurements (SUCRA: 99.7%), contrasting with minimal therapeutic benefit from low-frequency electrical stimulation (LFES, SUCRA: 0.8%). The therapeutic hierarchy for Functional Ambulation Category enhancement, per SUCRA metrics, was: tDCS (99.7%) > FES-Cycling (82.0%) > 4-ch FES (68.1%) > multi-ch FES (60.2%) > FES (51.9%) = TEAS+tDCS (51.9%) > NEMS (50.6%) > usual care (UC, 18.3%) > 2-ch FES (16.5%) > LFES (0.8%).

### 3.6 Adverse reactions

The included studies reported relatively few adverse events. A single study reported 7 incidents of falls and one case of femoral fracture in the functional electrical stimulation (FES) cohort following intervention, whereas the control group exhibited 2 falls. One publication indicated that 2 participants in the tDCS group and 2 in the sham stimulation group reported pruritus, concurrently 2 control subjects described paresthesia. Overall, electrical stimulation demonstrated a favorable safety profile.

### 3.7 Publication bias

**Figure 5:**
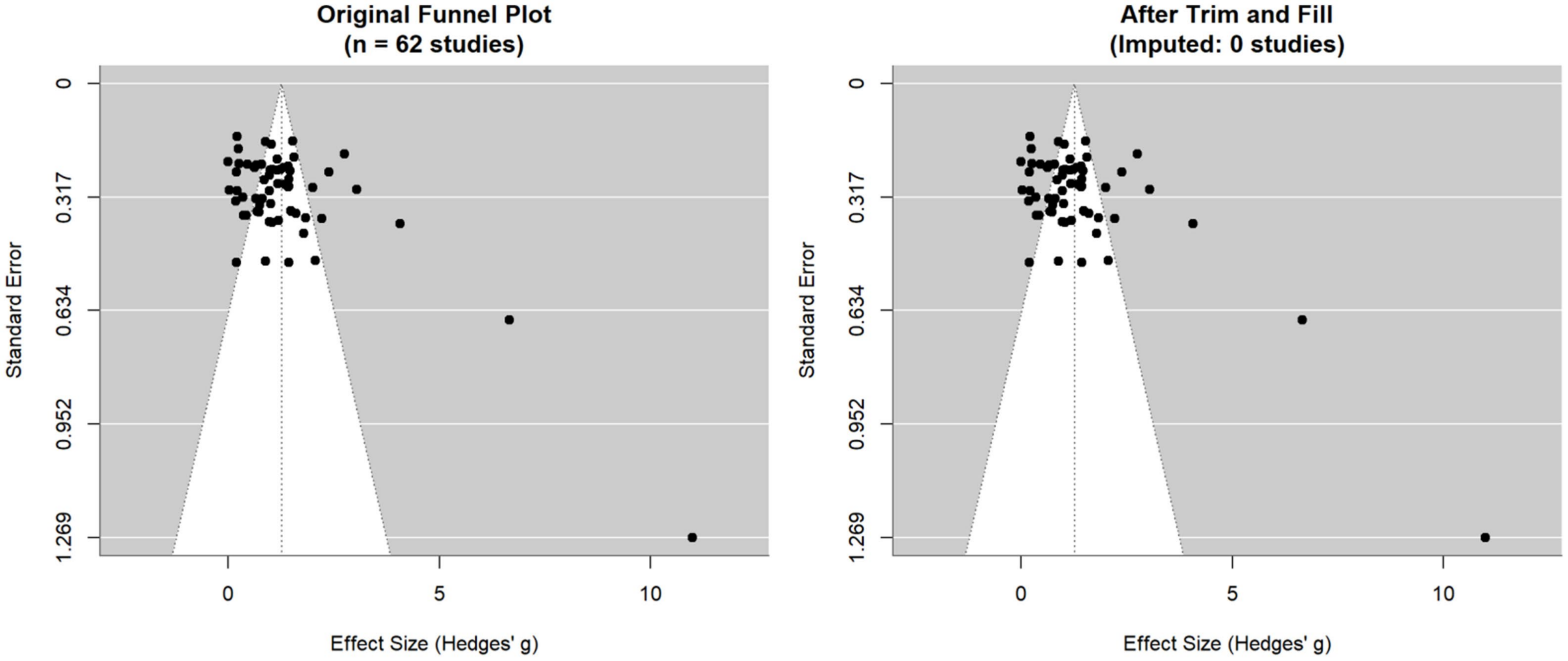
Comparison of the funnel plot for FMA-L score following trim-and-fill analysis.

**Figure 6:**
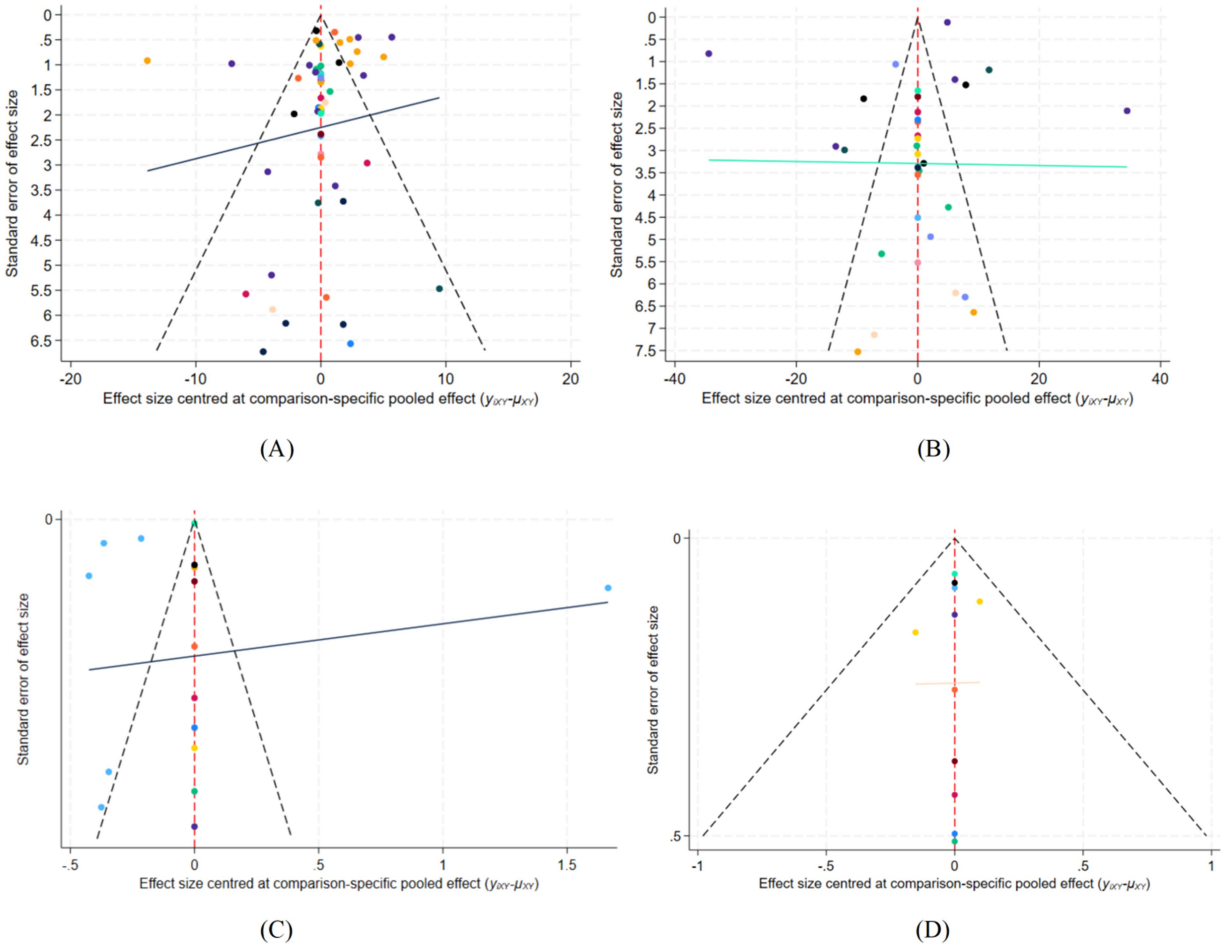
The funnel plot for all outcomes. **(A)** BBS score; **(B)** MBI score; **(C)** 10MWT; **(D)** FAC score.

To comprehensively evaluate potential publication bias, we applied an integrated analytic framework for outcomes with at least 10 contributing studies. This approach combined contour-enhanced funnel plots, Egger and Begg tests, and the trim-and-fill procedure. For the FMA-L outcome, the results were discordant: Egger’s test (*p* = 0.010), Begg’s test (*p* = 0.045), and the contour-enhanced funnel plot all suggested a risk of publication bias, whereas the trim-and-fill method did not impute any missing studies. This inconsistency may reflect the substantial heterogeneity within the evidence network, indicating that the observed funnel plot asymmetry likely reflects a mixture of true variability and potential bias (Figure 5). By contrast, all other outcomes (BBS: *p* = 0.390/0.220; MBI: *p* = 0.105/0.186; 10MWT: *p* = 0.861/0.681; FAC: *p* = 0.618/0.920) showed non-significant test results and displayed symmetrical funnel plots, collectively indicating a low risk of publication bias (Figure 6). Regarding the implications for the NMA, the effect estimate for FMA-L should be interpreted with caution; however, any potential bias pertains only to a subset of the evidence network. The consistently low risk of bias across the remaining key outcomes provides robust support for the SUCRA-based ranking results. Taken together, although publication bias may attenuate the precision of the FMA-L estimates, it does not overturn the overarching conclusions regarding the relative effectiveness of the evaluated interventions.

## 4 Discussion

This study included 85 RCTs, involving 18 electrical stimulation modalities and 23 distinct stimulation protocols. Departing from conventional meta-analytical approaches, this investigation implemented network meta-analysis via Stata 18.0 and R 4.4.3 to holistically assess 23 electrical stimulation protocols across 18 modalities, evaluating efficacy/safety for post-stroke lower limb dysfunction through validated outcome measures (FMA-L, BBS, MBI, 10MWT, FAC). The FMA-L instrument quantitatively assesses limb motor control proficiency, with particular emphasis on evaluating the integrity of isolated joint movements, movement coordination, and reflex-mediated motor integration as indicators of neuromotor quality. The BBS scoring system monitors static and dynamic balance capabilities, providing critical data for fall risk prediction and balance training. The MBI provides a comprehensive assessment of ADL, with particular emphasis on quantifying autonomy during functional transfers, ambulation, and stair negotiation. Its aggregate score constitutes a pivotal metric for determining self-care capacity and discharge readiness. The 10MWT objectively quantifies functional walking speed, while the FAC assesses adaptability to different walking environments.

Based on SUCRA value analysis, EMG-FES combined with conventional FES demonstrated optimal efficacy in improving lower limb motor function in stroke patients (SUCRA = 89.0%). Its superior therapeutic effect stems from a synergistic “proactive neural drive + targeted functional remodeling” progressive rehabilitation pathway. EMG-FES establishes a closed-loop feedback system by detecting residual electromyographic signals to trigger electrical stimulation. The system operates via real-time synchronization of efferent motor commands with afferent proprioceptive feedback, conforming to Hebbian plasticity mechanisms that potentiate neural circuit reorganization through temporally correlated activation (33–36). Conventional FES precisely activates paralyzed or weakened lower limb muscles through electrical stimulation, inducing contractions that simulate natural movement patterns. This stimulation enhances motor unit recruitment and improves muscle co-contraction capacity, thereby optimizing key gait components (37, 38). EMG-FES establishes the neurophysiological substrate for recovery through neural drive mechanisms, while conventional FES concurrently enables precision neuromuscular remodeling and functional re-education. This dual synergy of neural reconstruction and motor control optimization collectively drives significant functional restoration, offering a novel rehabilitation strategy for post-stroke motor dysfunction. NMA indicated Multi-ch FES yielded significantly greater therapeutic efficacy for gait balance rehabilitation (SUCRA = 85.6%) compared to conventional single-channel FES (SUCRA = 55.7%). As an innovative rehabilitation technology, Multi-ch FES utilizes independently controlled electrode channels to deliver synchronous or sequential electrical stimulation, activating multiple lower limb muscle groups for synergistic neuromuscular optimization (39). The intervention employs spatiotemporal precision modulation: spatial targeting of multiple neuromuscular sites coupled with temporally phased stimulation synchronized to gait phase transitions, achieving physiological rhythm fidelity (40–42). Compared to traditional single-channel FES, the multi-channel approach designs stimulation based on muscle synergy patterns, more naturally simulating physiological gait while improving both immediate and long-term kinematic parameters (43). Despite identical SUCRA values (71.9%), CCNEMS exhibited a substantially higher probability of being the optimal intervention (PreBest = 25.9%) compared to LFES (PreBest = 6.2%), confirming its statistical superiority across ranking metrics. Thus, CCNEMS shows SUCRA and optimal treatment probability (PreBest), establishing it as the optimal protocol for improving MBI in stroke patients with lower limb dysfunction. CCNEMS represents an advanced NEMS technology that induces targeted skeletal muscle contraction through precisely regulated electrical pulses (44, 45). The paradigm-shifting innovation resides in programmable current parameters that dynamically simulate physiological neural firing patterns, facilitating optimal neuroplastic adaptation (46). In summary, CCNEMS utilizes controlled electrical parameters to deliver neuromuscular stimulation, designed to enhance therapeutic efficacy, minimize adverse effects, and promote co-adaptation of central and peripheral neural systems (47, 48). LFES is a significant neuromuscular rehabilitation technique. It delivers low-frequency electrical currents (typically 1–100 Hz) through surface electrodes to stimulate target neuromuscular tissues, aiming to elicit muscle contractions or modulate neural activity for functional improvement. LFES employs periodic pulsed currents (1–100 Hz) delivered transcutaneously to activate motor neurons (49, 50), effectively generating functional contractions in paralyzed muscles through direct stimulation of motor nerves or muscle fibers. Optimal efficacy occurs at 20–30 Hz (51), balancing effective muscle activation with minimal fatigue induction while demonstrating superior patient tolerance compared to higher-frequency protocols (49). Additionally, LFES specifically addresses gait abnormalities like foot drop and propulsion deficits. It potentiates neuroplasticity through enhanced corticospinal excitability and functional reorganization of motor cortical areas, establishing central mechanisms for sustainable gait rehabilitation (52–55). These synergistic mechanisms collectively enhance walking ability, evidenced by optimized 10MWT performance including increased speed, improved gait patterns, and enhanced endurance. Robust statistical confirmation (SUCRA = 66.2%) demonstrates LFES’s significant efficacy in enhancing ambulatory velocity as measured by the 10MWT. tDCS is a non-invasive brain stimulation technique proven to be a promising neurorehabilitation intervention (56). Its primary mechanism involves applying weak direct current (typically 1–2 mA) through scalp electrodes to modulate neurogenic excitability networks in both affected and unaffected hemispheres post-stroke (57–59). By regulating cortical excitability via low-intensity currents, tDCS promotes neuroplasticity, with targeted modulation of motor and cerebellar cortices potentially improving lower limb motor function. Studies demonstrate that Anodal tDCS potentiates lesioned-hemisphere excitability, whereas cathodal tDCS suppresses pathological contralateral hyperactivity, remodeling corticospinal transmission via LTP mechanisms while activating cerebellar-cortical pathways for motor coordination optimization (60–62). The exceptional SUCRA value (99.7%) derived from FAC assessment provides definitive statistical evidence of tDCS’s absolute superiority as the optimal intervention for post-stroke lower limb rehabilitation.

This study has several limitations. The majority of included studies omitted reporting on participant and outcome assessor blinding procedures, creating potential performance and detection bias that compromises methodological rigor. Significant inter-study variations in stimulation parameters (intensity/location) represent a probable source of clinical and methodological heterogeneity. Constraints of original studies precluded detailed subgroup analysis of disease duration and treatment cycles. Insufficient blinding implementation in most trials may have increased bias risk. Limiting to English/Chinese studies could overlook important evidence, and statistical patterns suggest negative results might be missing from the analysis. Future large-scale, high-quality studies are needed to validate these findings.

## 5 Conclusion

The extant literature substantiates that electromagnetic stimulation yields statistically and clinically significant improvements across core rehabilitation domains: limb motor control (FMA-L), static/dynamic balance (BBS), activities of daily living (MBI), functional gait speed (10MWT), and ambulatory capacity (FAC) in post-stroke lower limb dysfunction. Methodological constraints and sample size limitations necessitate future investigation through rigorously designed trials featuring multimodal outcome batteries and methodologically robust multi-center randomized controlled trials.

## Data Availability

The original contributions presented in the study are included in the article/Supplementary material, further inquiries can be directed to the corresponding author.

## Glossary

ADL: Activities of daily living
NMA: Network meta-analysis
PreBest: Optimal treatment probability
SUCRA: Surface under the cumulative ranking analysis
MD: Mean difference
95%CI: 95% confidence intervals
REM: Random-EFFECTS MODELS
MeSH: Medical subject headings
RCTs: Randomized Controlled Trials
FMA-L: Fugl-Meyer assessment for lower extremity
BBS: Berg balance scale
MBI: Modified Barthel index
10MWT: 10-Meter walk test
FAC: Functional ambulation category
FES: Functional electrical stimulation
4-ch FES: Quad-channel FES
2-ch FES: Dual-channel FES
Multi-ch FES: Multi-channel FES
tDCS: Transcranial direct current stimulation
rTMS: repetitive transcranial magnetic stimulation
TENS: Transcutaneous electrical nerve stimulation
TEAS: Transcutaneous acupoint electrical stimulation
NEMS: Neuromuscular electrical stimulation
LFES: Low-frequency electrical stimulation
IFES: Intermediate-frequency electrical
DFES: Deep muscle electrical stimulation
CCNEMS: Controllable current neuromuscular electrical stimulation
FNS: Fastigial nucleus stimulation
MJL-WFES: Multi-joint linked wearable FES
EMG: Muscle-triggered electrical stimulation
taVNS: Transcutaneous auricular vagus nerve stimulation
