# Supplementary material for: Network meta-analysis of different electrical stimulation therapies for lower limb functional rehabilitation in stroke patients
Source: Front Neurol. 2026 Jan 12;16:1682671. doi: 10.3389/fneur.2025.1682671 (PMC12833568; doi:10.3389/fneur.2025.1682671)
Supplement: Supplementary file 2 [file Table_2.docx]

Table 1,The search strategy for the respective database.

| Database name | Search strategies |
| --- | --- |
| CNKI | (SU%='电刺激' ) AND (SU%='卒中' + '脑卒中' + '中风' + '脑中风' + '脑血管意外' + '急性脑血管意外' + '急性中风' + '急性卒中' + '脑血管中风' + 'CVA(脑血管意外)' + 'CVAs(脑血管意外)' + '急性脑卒中') AND (SU%='下肢' + '运动' + '下肢功能康复' + '步态' + '步行功能' + '平衡' ) AND (SU%='随机分配' + '随机对照试验' + '随机化试验' + '随机分组' + '随机实验' + '随机对照研究' + '临床试验' + 'RCT') |
| Wanfang | 主题:(电刺激) and 主题:(卒中 or 脑卒中 or 脑血管意外 or 中风 or 脑中风 or 急性脑血管意外 or 急性脑卒中 or 急性卒中 or 急性中风 or CVAs(脑血管意外) or CVA(脑血管意外) or 脑血管中风) and 题名或关键词:(下肢 or 运动 or 步态 or 平衡 or 下肢功能康复 or 步行功能) and 主题:(随机分配 or 随机对照试验 or 随机分组 or 临床试验 or 随机化对照试验 or 随机对照研究 or 随机试验) |
| VIP | (M=电刺激) AND (M=(中风 OR 脑卒中 OR 缺血性脑卒中 OR 脑血管意外 OR 脑中风 OR 急性脑血管意外 OR 急性卒中 OR 急性脑卒中 OR 急性中风 OR 卒中)) AND (M=(运动 OR 步态 OR 平衡 OR 下肢功能康复 OR 步行功能 OR 下肢)) AND (R=(随机对照 OR 随机分组 OR 随机 OR rct OR RCT OR 随机对照试验 OR 临床试验 OR 随机分配 OR 随机化对照试验 OR 随机对照研究)) |
| PubMed | #1：((((((Stimulation, Electric) OR (Electric Stimulations)) OR (Stimulations, Electric)) OR (Electrical Stimulation)) OR (Electrical Stimulations)) OR (Stimulation, Electrical)) OR (Stimulations, Electrical)  #2： ((((((((((((((((((((strokes) OR (cerebrovascular accident)) OR (cerebrovascular accidents)) OR (cerebral stroke)) OR (cerebral strokes)) OR (stroke, cerebral)) OR (cerebrovascular apoplexy)) OR (Apoplexy, cerebrovascular)) OR (vascular accident,brain)) OR (brain vascular accident)) OR (brain vascular accidents)) OR (Stroke,cerebrovascular)) OR (Strokes,cerebrovascular)) OR (apoplexy)) OR (CVA(cerebrovascular accident))) OR (CVAs (cerebrovascular accident))) OR (Acute cerebrovascular accident)) OR (Acute cerebrovascular accidents)) OR (Stroke, acute)) OR (acute stroke)) OR (acute strokes)  #3： (((((((gait) OR (walking)) OR (lower extremity)) OR (lower limb)) OR (lower limbs)) OR (ambulation)) OR (membrum inferius)) OR (locomotion)  #4：((((((((((random allocation) OR (placebos)) OR (clinical trials, randomized)) OR (trials, randomized clinical)) OR (controlled clinical trials, randomized)) OR (randomized)) OR (randomised)) OR (randomly)) OR (trial)) OR (phase)) OR (rct)  #5：(((#1) AND (#2)) AND (#3)) AND (#4) |
| Cochrane Library | #1: (Electric Stimulations):ti,ab,kw OR (Stimulation, Electric):ti,ab,kw OR (Stimulations, Electrical):ti,ab,kw OR (Stimulations, Electric):ti,ab,kw OR (Electrical Stimulations):ti,ab,kw OR (Electrical Stimulation):ti,ab,kw OR (Stimulation, Electrical):ti,ab,kw  #2: (strokes):ti,ab,kw OR (CVA (Cerebrovascular Accident)):ti,ab,kw OR (Apoplexy):ti,ab,kw OR (Cerebrovascular Accident):ti,ab,kw OR (Cerebrovascular Stroke):ti,ab,kw OR (ascular Accident, Brain):ti,ab,kw OR (Brain Vascular Accidents):ti,ab,kw OR (Cerebrovascular Strokes):ti,ab,kw OR (Stroke, Cerebrovascular):ti,ab,kw OR (Cerebral Strokes):ti,ab,kw OR (Vascular Accidents, Brain):ti,ab,kw OR (Strokes, Cerebral):ti,ab,kw OR (Cerebrovascular Accidents):ti,ab,kw OR (Strokes, Cerebrovascular):ti,ab,kw OR (Brain Vascular Accident):ti,ab,kw OR (Cerebral Stroke):ti,ab,kw OR (Cerebrovascular Apoplexy):ti,ab,kw OR (Stroke, Cerebral):ti,ab,kw OR (Apoplexy, Cerebrovascular):ti,ab,kw OR (CVAs (Cerebrovascular Accident)):ti,ab,kw OR (Cerebrovascular Accidents, Acute):ti,ab,kw OR (Cerebrovascular Accident, Acute):ti,ab,kw OR (Acute Strokes):ti,ab,kw OR (Acute Stroke):ti,ab,kw OR (Acute Cerebrovascular Accidents):ti,ab,kw OR (Acute Cerebrovascular Accident):ti,ab,kw OR (Stroke, Acute):ti,ab,kw OR (Strokes, Acute):ti,ab,kw  #3: (gait):ti,ab,kw OR (walking):ti,ab,kw OR (lower extremity):ti,ab,kw OR (lower limb):ti,ab,kw OR (lower limbs):ti,ab,kw OR (ambulation):ti,ab,kw OR (membrum inferius):ti,ab,kw OR (locomotion):ti,ab,kw  #4：(random allocation):ti,ab,kw OR (controlled clinical trials, randomized):ti,ab,kw OR (randomized):ti,ab,kw OR (randomised):ti,ab,kw OR (randomly):ti,ab,kw OR (trial):ti,ab,kw OR (phase):ti,ab,kw OR (rct):ti,ab,kw  #5: #1 AND #2 AND #3 AND #4 |
| Web of Science | #1: ((((((TS=(Stimulation, Electric )) OR TS=( Electric Stimulations )) OR TS=(Stimulations, Electric )) OR TS=( Electrical Stimulation)) OR TS=(Electrical Stimulations)) OR TS=(Stimulation, Electrical)) OR TS=(Stimulations, Electrical) and Preprint Citation Index (Exclude – Database)  #2: ((((((((((((((((((((TS=(strokes)) OR TS=(cerebrovascular accident)) OR TS=(cerebrovascular accidents)) OR TS=(cerebral stroke)) OR TS=(cerebral strokes)) OR TS=(stroke, cerebral )) OR TS=(cerebrovascular apoplexy)) OR TS=(Apoplexy, cerebrovascular)) OR TS=(vascular accident,brain)) OR TS=(brain vascular accident)) OR TS=(brain vascular accidents)) OR TS=(Stroke,cerebrovascular)) OR TS=(Strokes,cerebrovascular)) OR TS=(apoplexy )) OR TS=(CVA(cerebrovascular accident))) OR TS=(CVAs (cerebrovascular accident))) OR TS=(Acute cerebrovascular accident )) OR TS=(Acute cerebrovascular accidents)) OR TS=(Stroke, acute)) OR TS=(acute stroke)) OR TS=(acute strokes) and Preprint Citation Index (Exclude – Database)  #3: (((((((TS=(gait)) OR TS=(walking)) OR TS=( lower extremity)) OR TS=( lower limb)) OR TS=(lower limbs)) OR TS=( ambulation)) OR TS=(membrum inferius)) OR TS=(locomotion) and Preprint Citation Index (Exclude – Database)  #4:((((((((((TS=(random allocation)) OR TS=(placebos)) OR TS=(clinical trials, randomized)) OR TS=(trials, randomized clinical)) OR TS=(controlled clinical trials, randomized)) OR TS=(randomized)) OR TS=(randomised)) OR TS=(randomly)) OR TS=(trial)) OR TS=(phase)) OR TS=(rct) and Preprint Citation Index (Exclude – Database)  #5: #1 AND #2 AND #3 AND #4 |
| Embase | #1： 'electric stimulation'/exp OR 'electric stimulation' OR (electric AND ('stimulation'/exp OR stimulation)) OR 'electrical stimulation':ti,ab,kw OR 'stimulation, electric':ti,ab,kw OR 'electro stimulation':ti,ab,kw OR electrostimulus:ti,ab,kw OR galvanostimulation:ti,ab,kw OR electrostimulation:ti,ab,kw  #2： 'brain vascular accident'/exp OR 'brain vascular accident' OR (('brain'/exp OR brain) AND vascular AND ('accident'/exp OR accident)) OR 'accident, cerebrovascular':ti,ab,kw OR 'acute cerebrovascular lesion':ti,ab,kw OR 'acute focal cerebral vasculopathy':ti,ab,kw OR 'apoplectic stroke':ti,ab,kw OR apoplexy:ti,ab,kw OR apoplexia:ti,ab,kw OR 'acute stroke':ti,ab,kw OR 'blood flow disturbance, brain':ti,ab,kw OR 'brain accident':ti,ab,kw OR 'brain attack':ti,ab,kw OR 'brain blood flow disturbance':ti,ab,kw OR 'brain insult':ti,ab,kw OR 'brain insultus':ti,ab,kw OR 'cerebral apoplexia':ti,ab,kw OR 'cerebral insult':ti,ab,kw OR 'cerebral stroke':ti,ab,kw OR 'cerebral vascular accident':ti,ab,kw OR 'cerebral vascular insufficiency':ti,ab,kw OR 'cerebro vascular accident':ti,ab,kw OR 'cerebrovascular arrest':ti,ab,kw OR 'cerebrovascular failure':ti,ab,kw OR 'cerebrovascular injury':ti,ab,kw OR 'cerebrovascular insufficiency':ti,ab,kw OR 'cerebrovascular insult':ti,ab,kw OR 'cerebrum vascular accident':ti,ab,kw OR 'cryptogenic stroke':ti,ab,kw OR 'insultus cerebralis':ti,ab,kw OR 'ischaemic seizure':ti,ab,kw OR 'ischemic seizure':ti,ab,kw OR stroke:ti,ab,kw OR 'thrombotic stroke':ti,ab,kw OR 'cerebrovascular accident':ti,ab,kw  #3： 'gait'/exp OR gait OR walking:ti,ab,kw OR 'lower extremity':ti,ab,kw OR 'lower limb':ti,ab,kw OR 'lower limbs':ti,ab,kw OR ambulation:ti,ab,kw OR 'membrum inferius':ti,ab,kw OR locomotion:ti,ab,kw  #4：'random allocation'/exp OR 'random allocation' OR (random AND allocation) OR 'controlled clinical trials, randomized':ti,ab,kw OR randomized:ti,ab,kw OR randomised:ti,ab,kw OR randomly:ti,ab,kw OR trial:ti,ab,kw OR phase:ti,ab,kw OR rct:ti,ab,kw  #5 ：#1 AND #2 AND # 3 AND #4 |

CNKI: China National Knowledge Infrastructure; WanFang: the WanFang Database; VIP: the Chinese Scientific Journals Full-Text Database;Embase Database; Excerpta Medica Database; WOS Database: Web of Science Database;PubMed Database: the PubMed Database.
