# Supplementary material for: Network meta-analysis of different electrical stimulation therapies for lower limb functional rehabilitation in stroke patients
Source: Front Neurol. 2026 Jan 12;16:1682671. doi: 10.3389/fneur.2025.1682671 (PMC12833568; doi:10.3389/fneur.2025.1682671)
Supplement: Supplementary file 3 [file Table_3.docx]

**Supplement**

Forest map and subgroup analysis


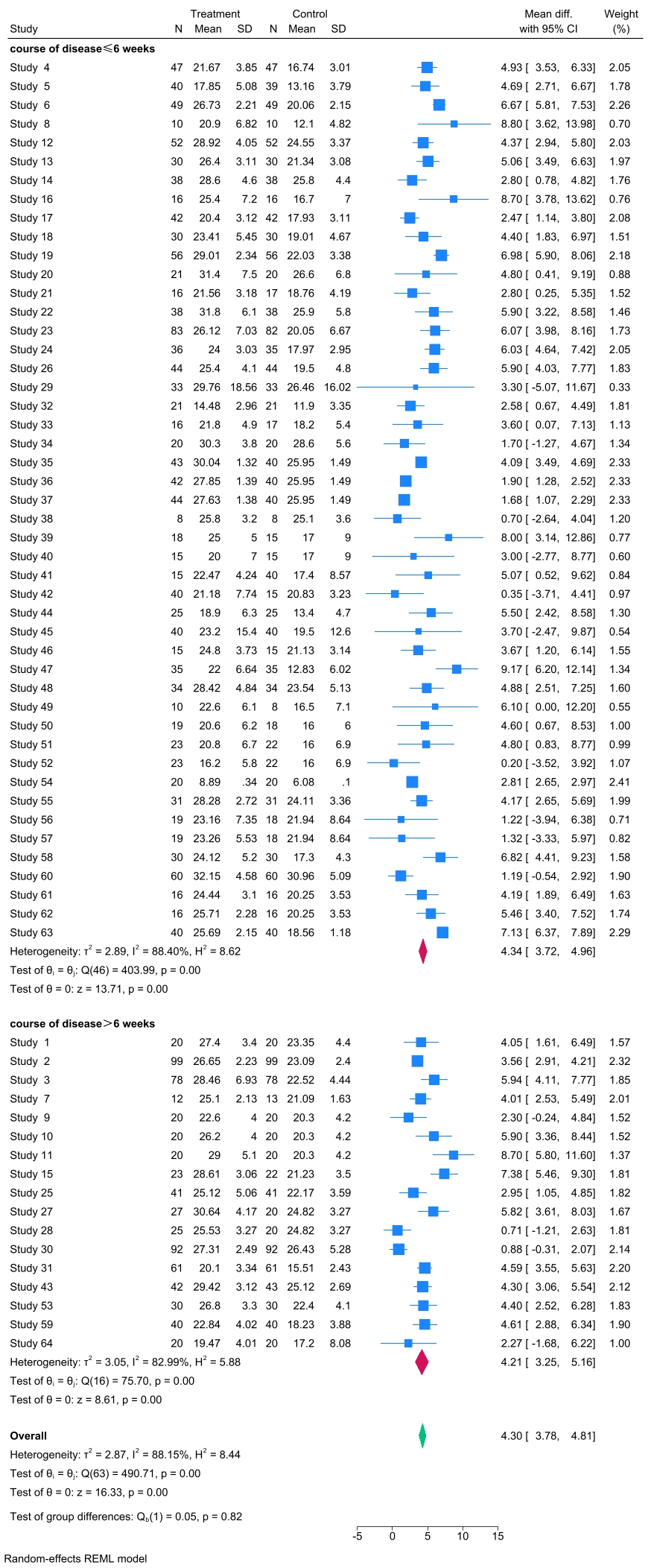


**Figure 1 Forest map and subgroup analysis: FMA-L score**


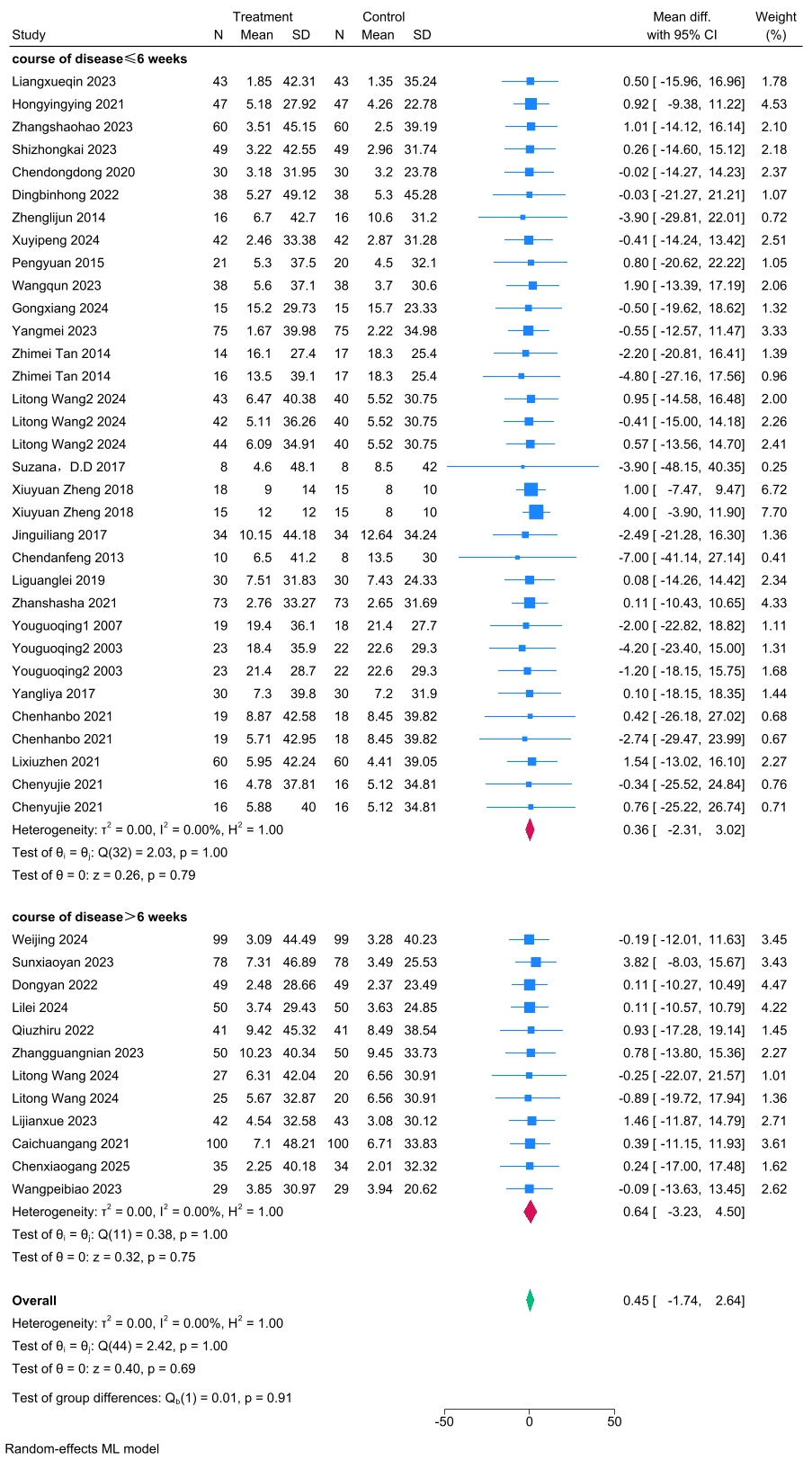


**Figure 2 Forest map and subgroup analysis: BBS score**

**
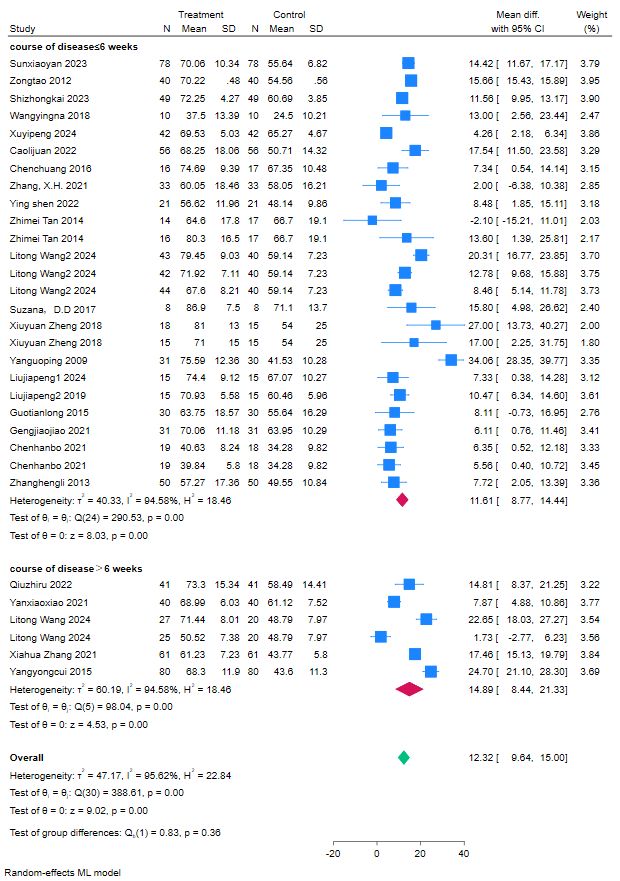
**

**Figure 3 Forest map and subgroup analysis: MBI score**

**
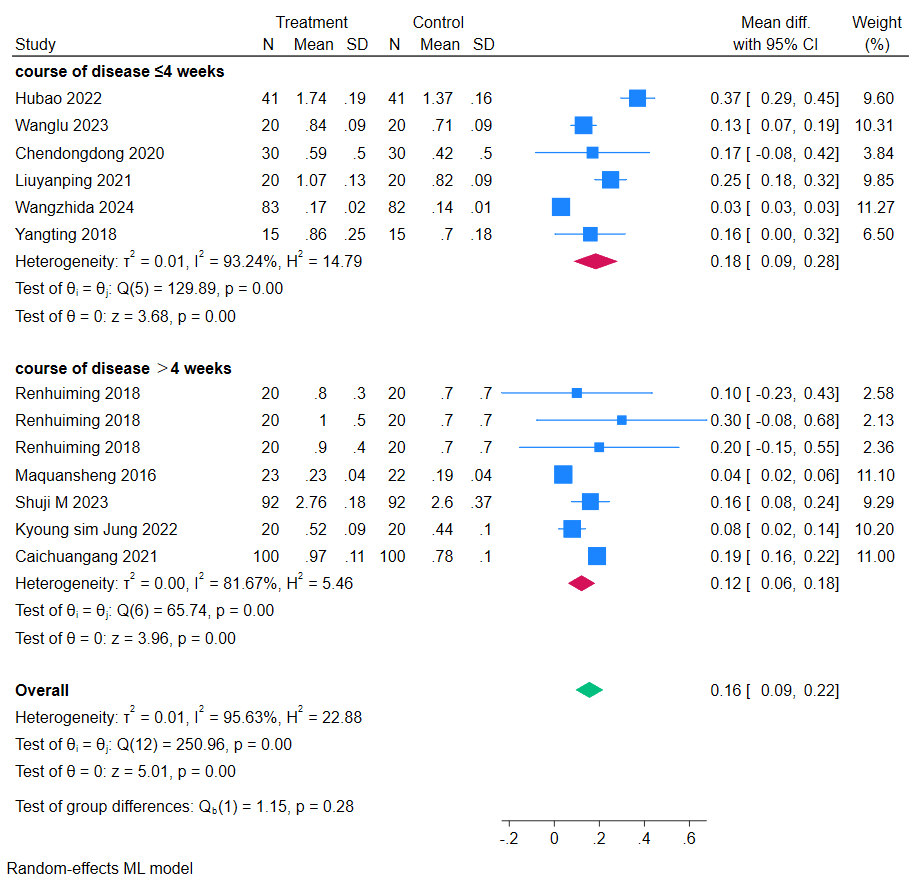
**

**Figure 4 Forest map and subgroup analysis: 10MWT**

**
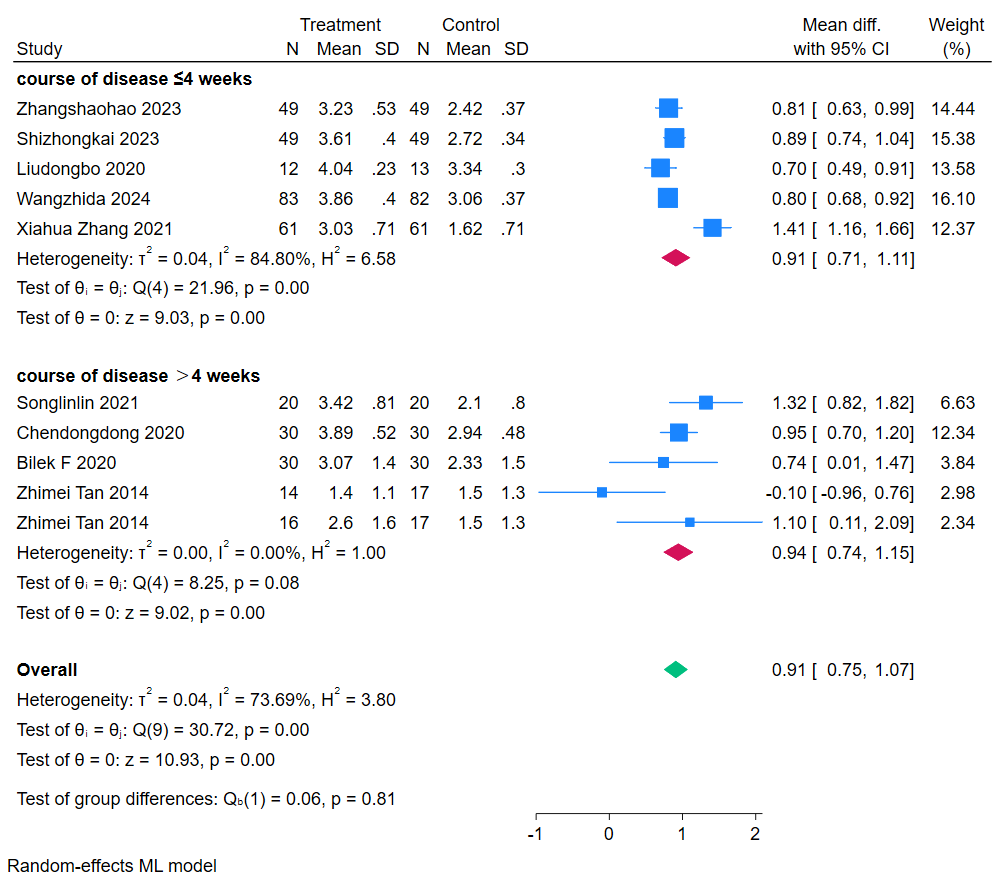
**

**Figure 5 Forest map and subgroup analysis: FAC score**
