# Supplementary material for: Network meta-analysis of different electrical stimulation therapies for lower limb functional rehabilitation in stroke patients
Source: Front Neurol. 2026 Jan 12;16:1682671. doi: 10.3389/fneur.2025.1682671 (PMC12833568; doi:10.3389/fneur.2025.1682671)
Supplement: Supplementary file 4 [file Table_4.docx]

**Supplement**

Trajectory diagram and density diagram


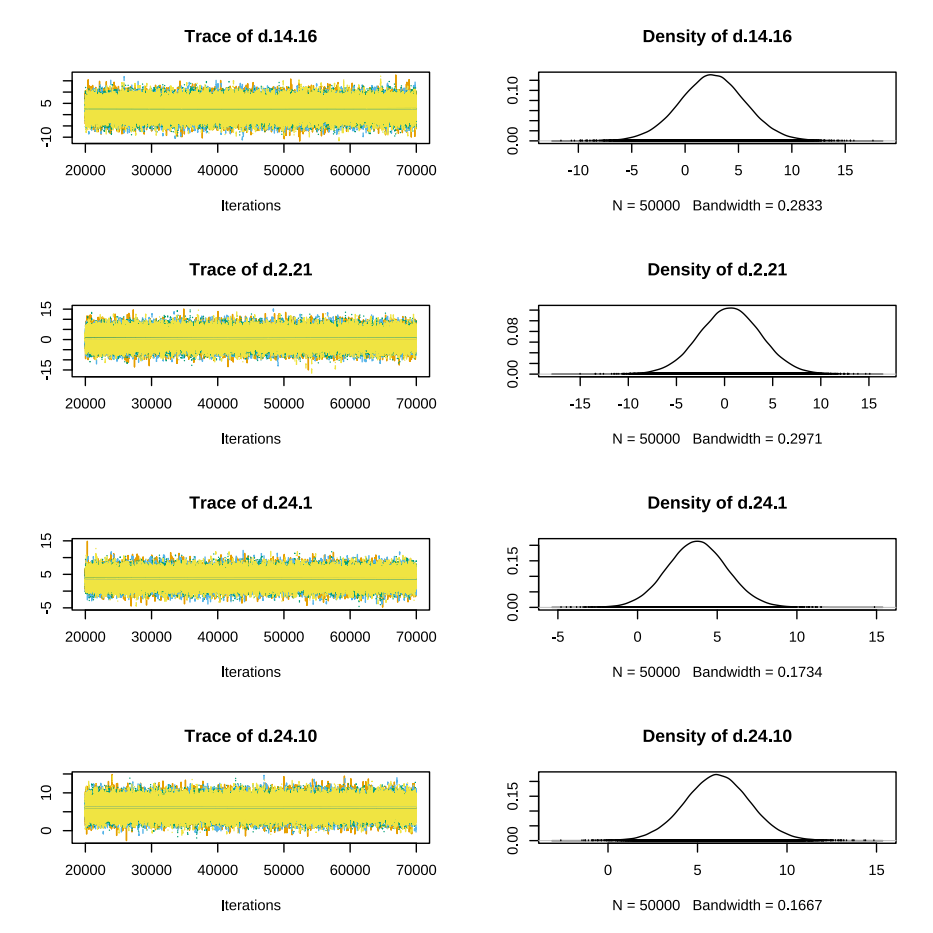

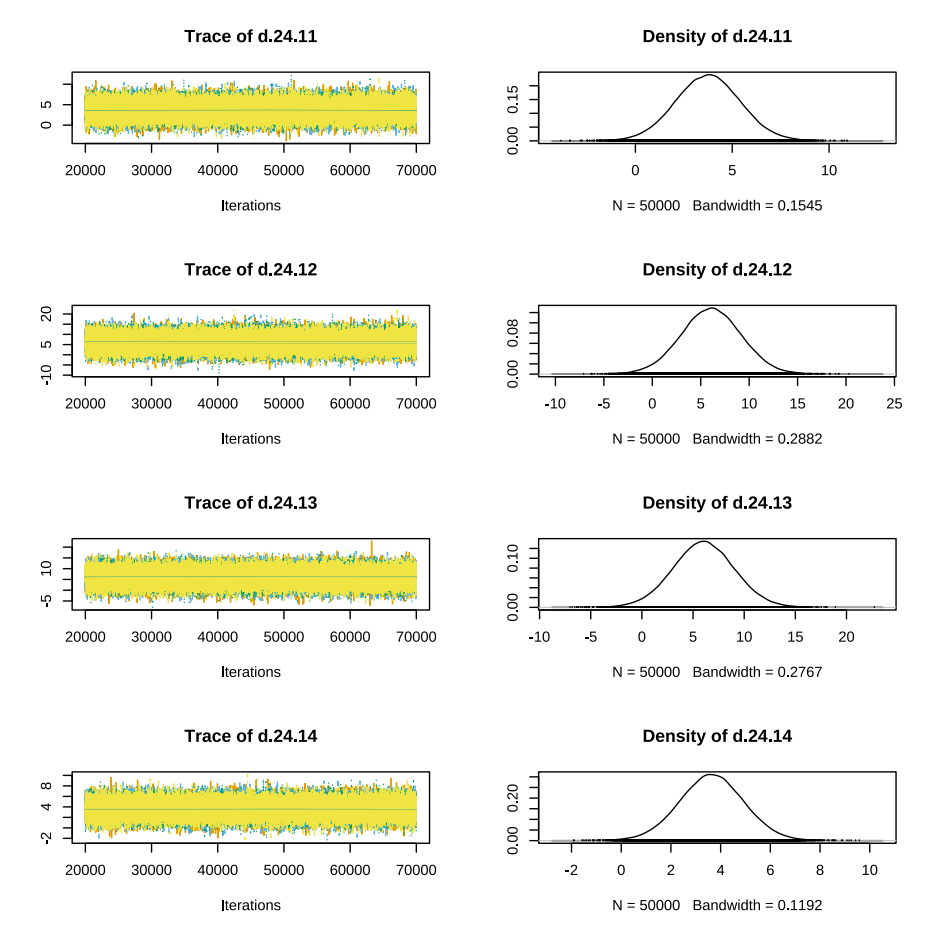

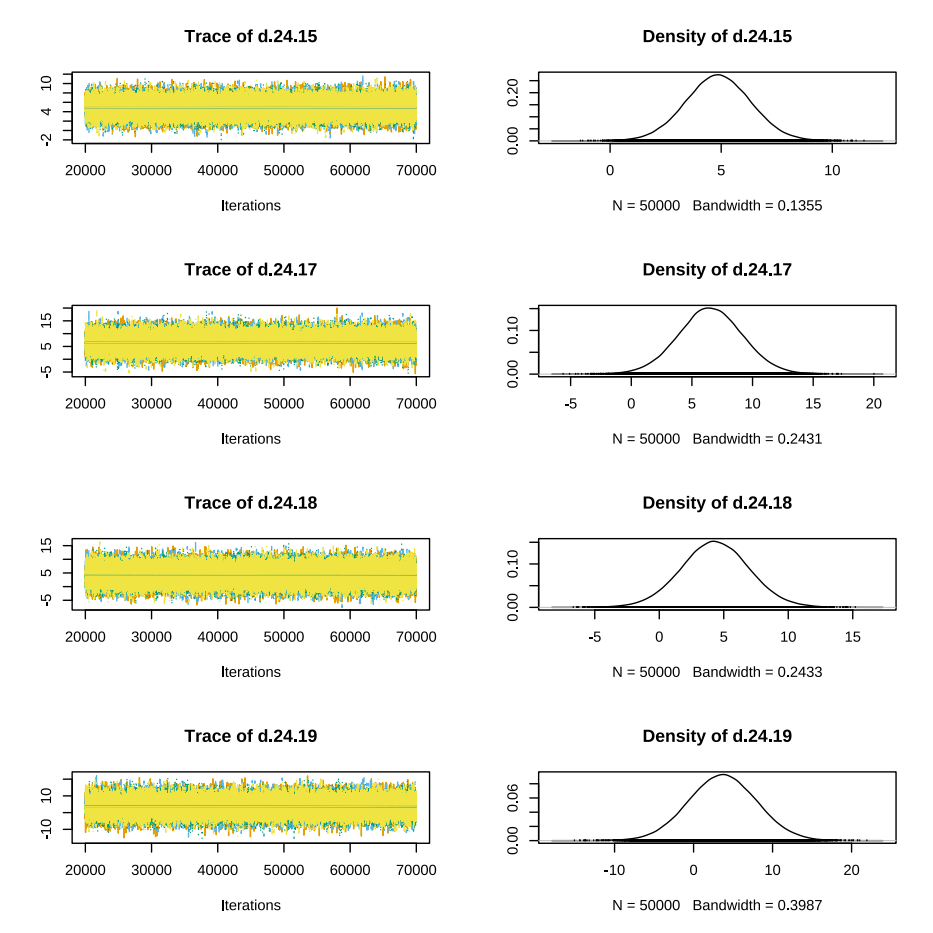

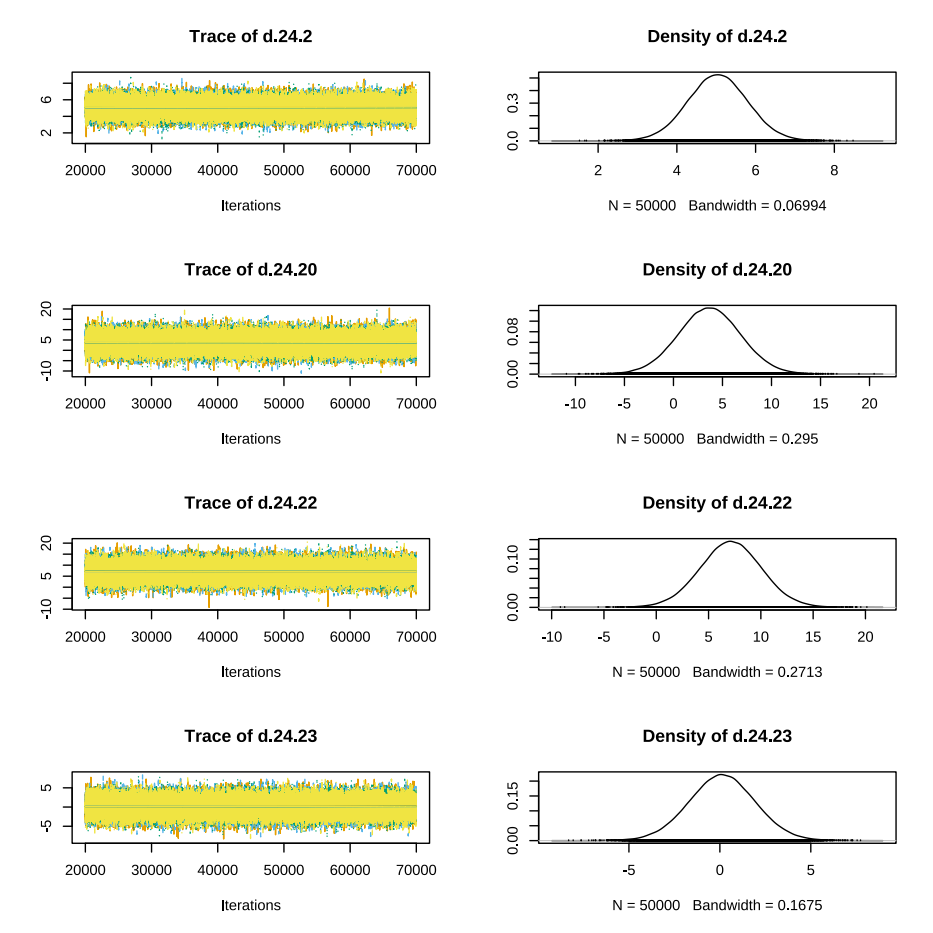

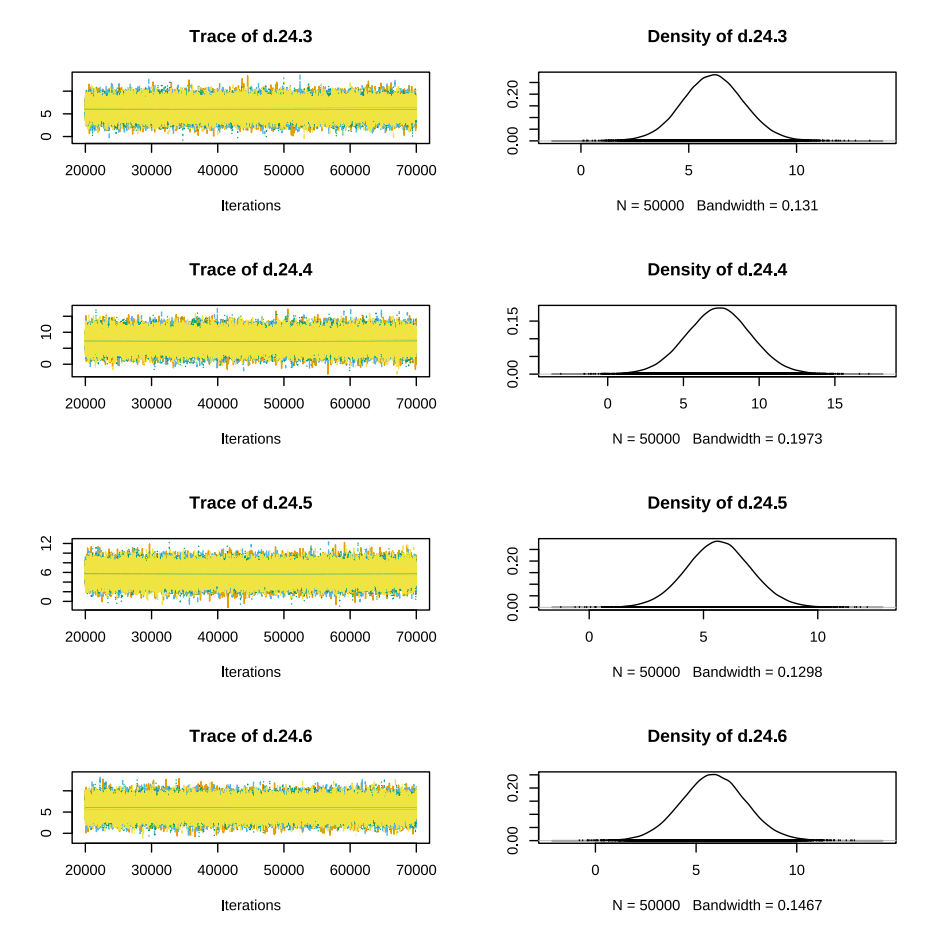

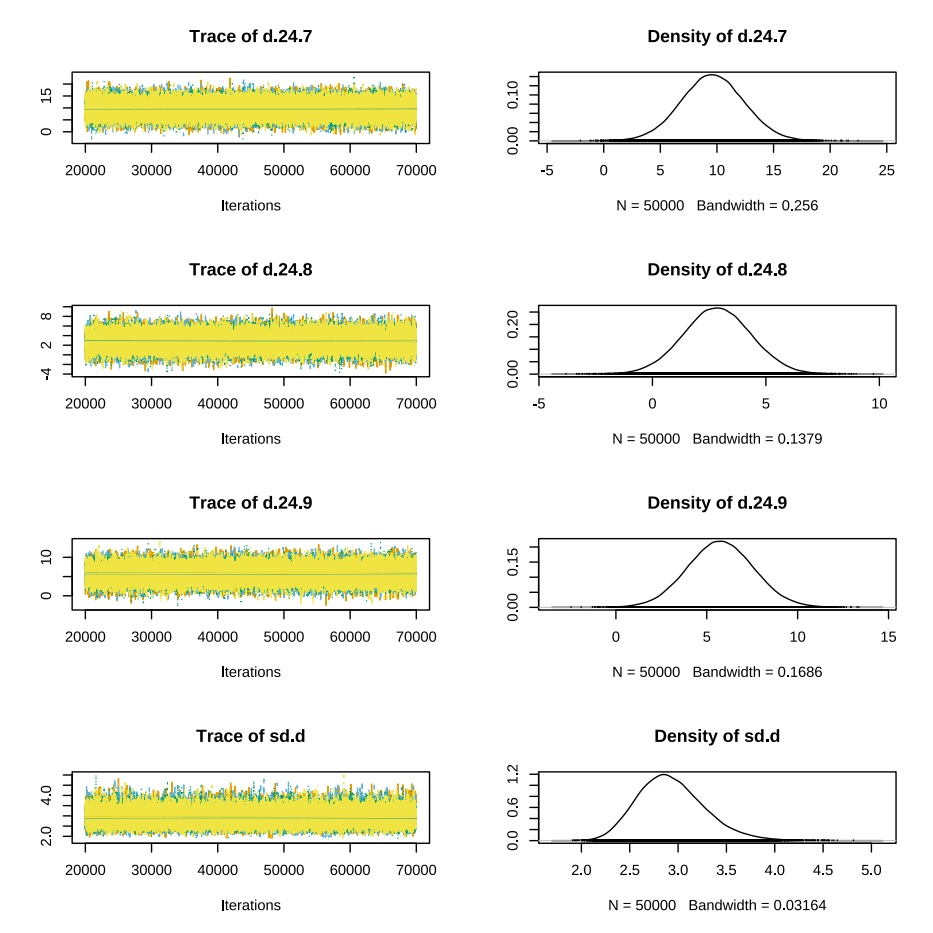


**Figure 1** **Trajectory diagram and density diagram: FMA score**


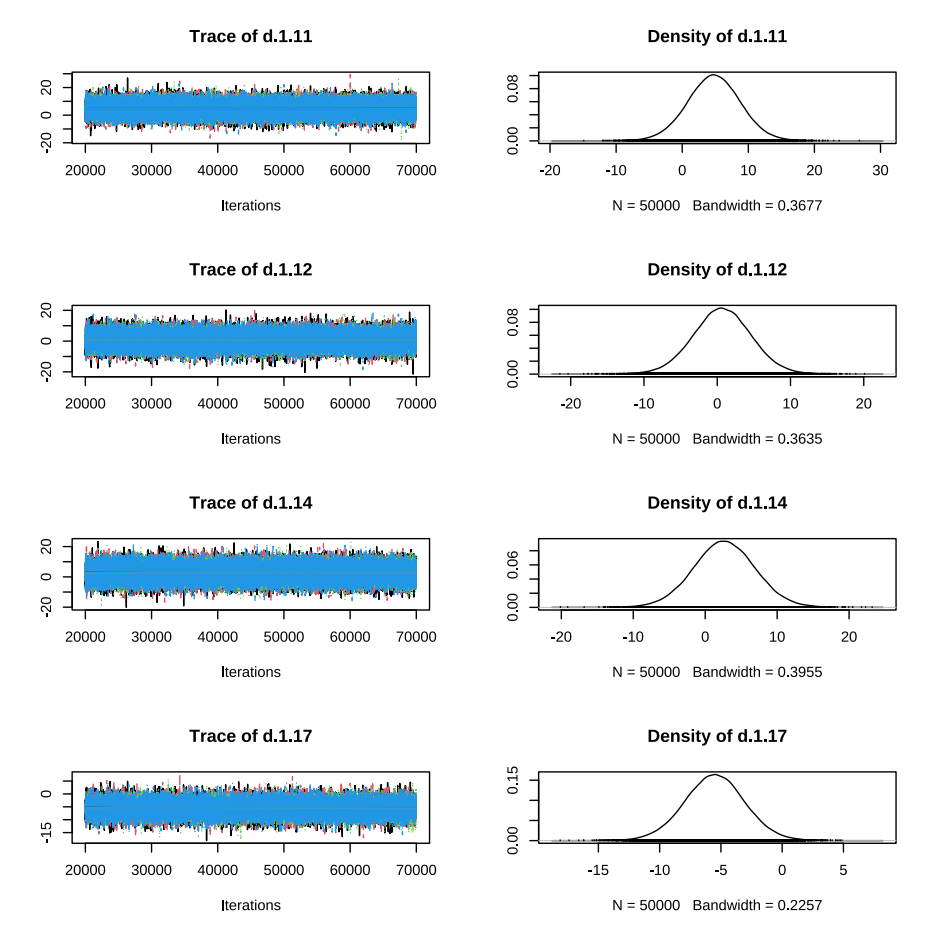

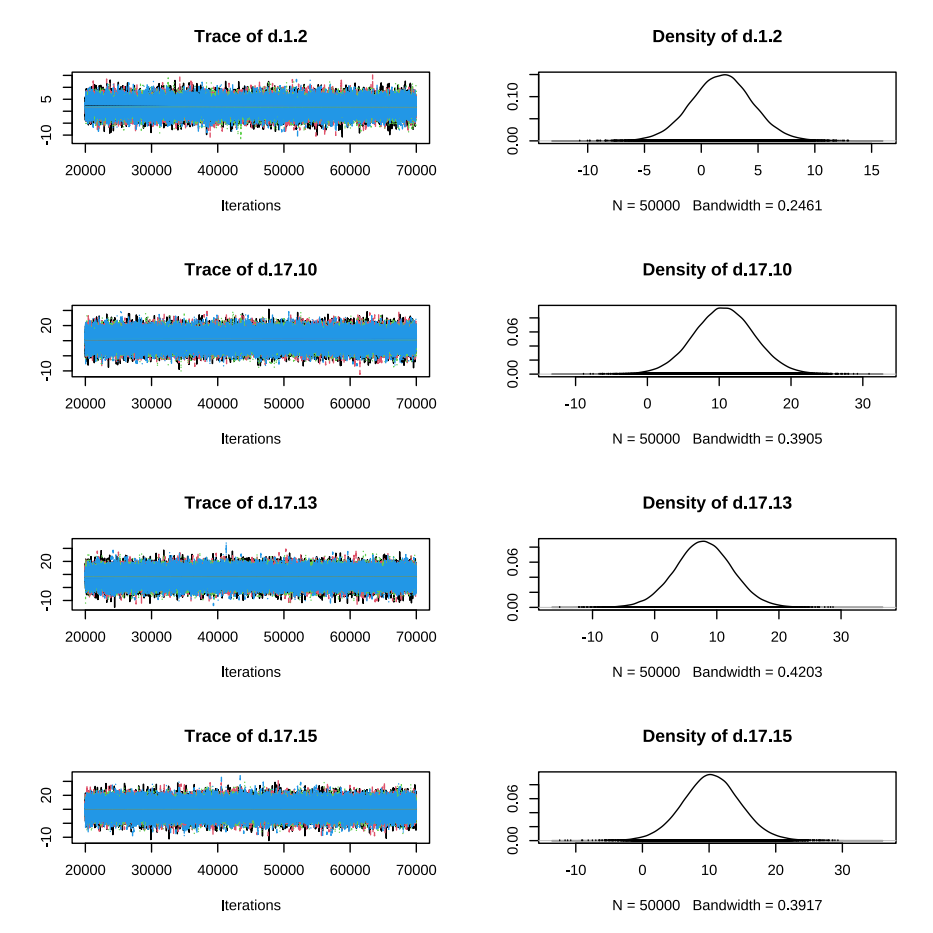

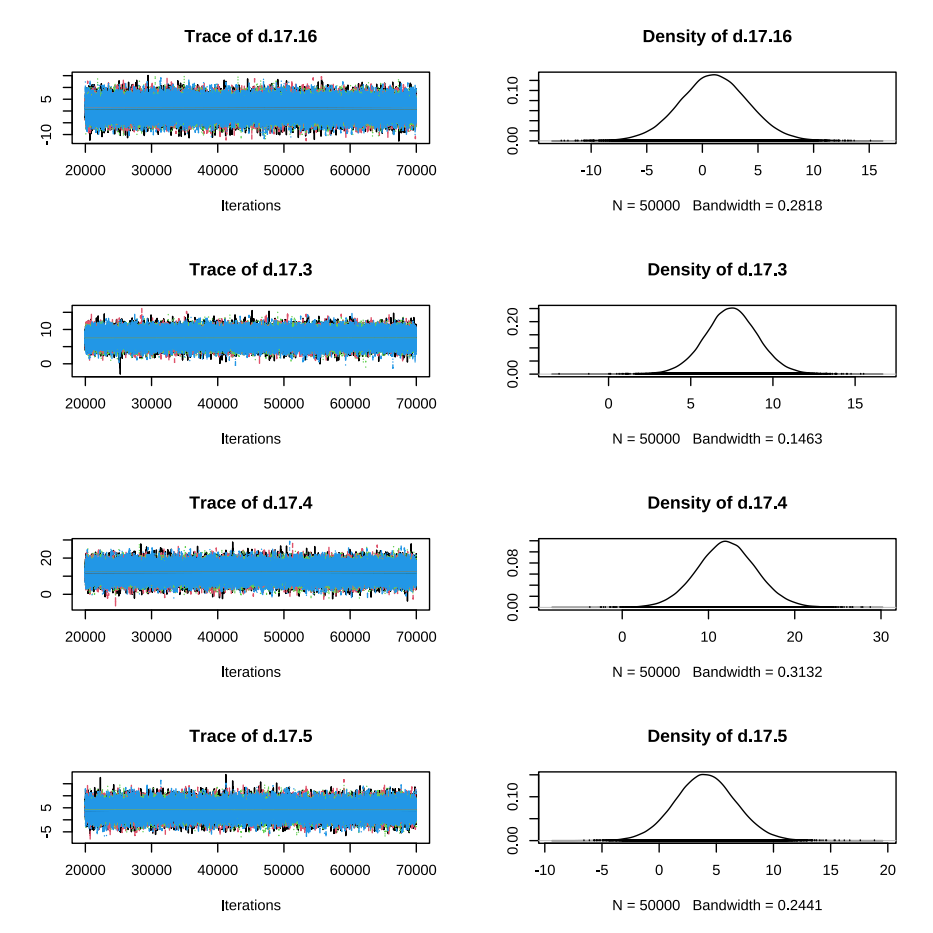

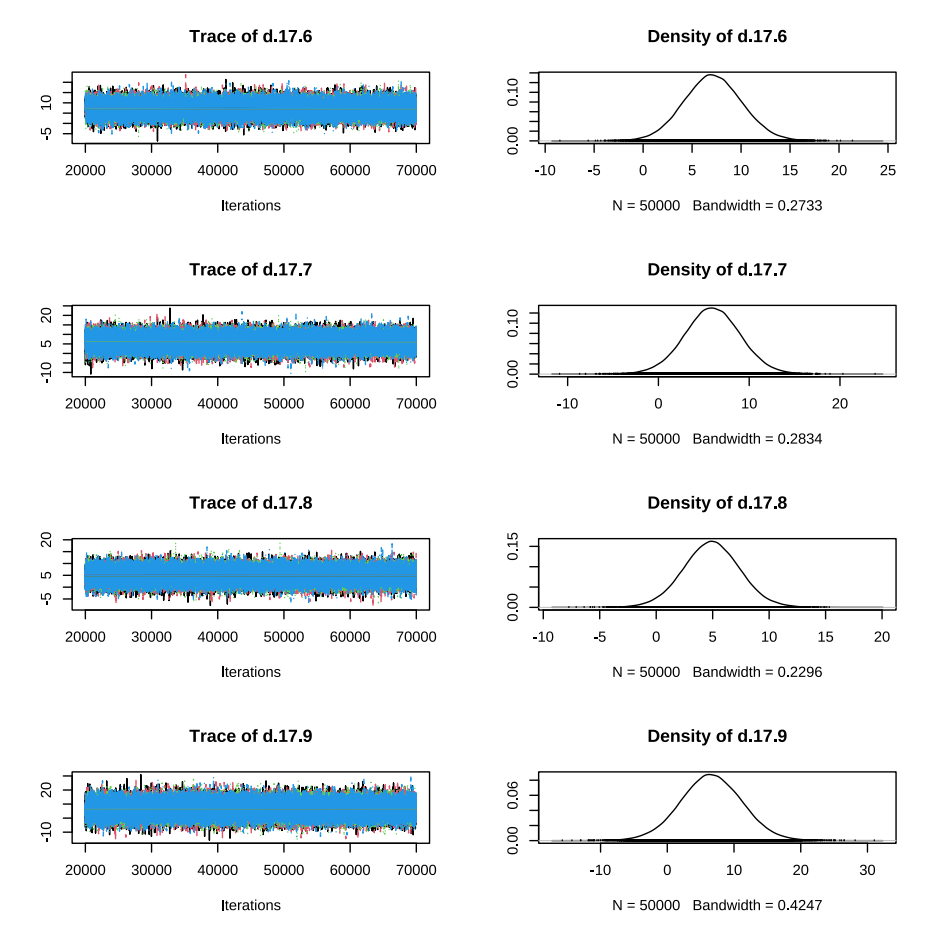

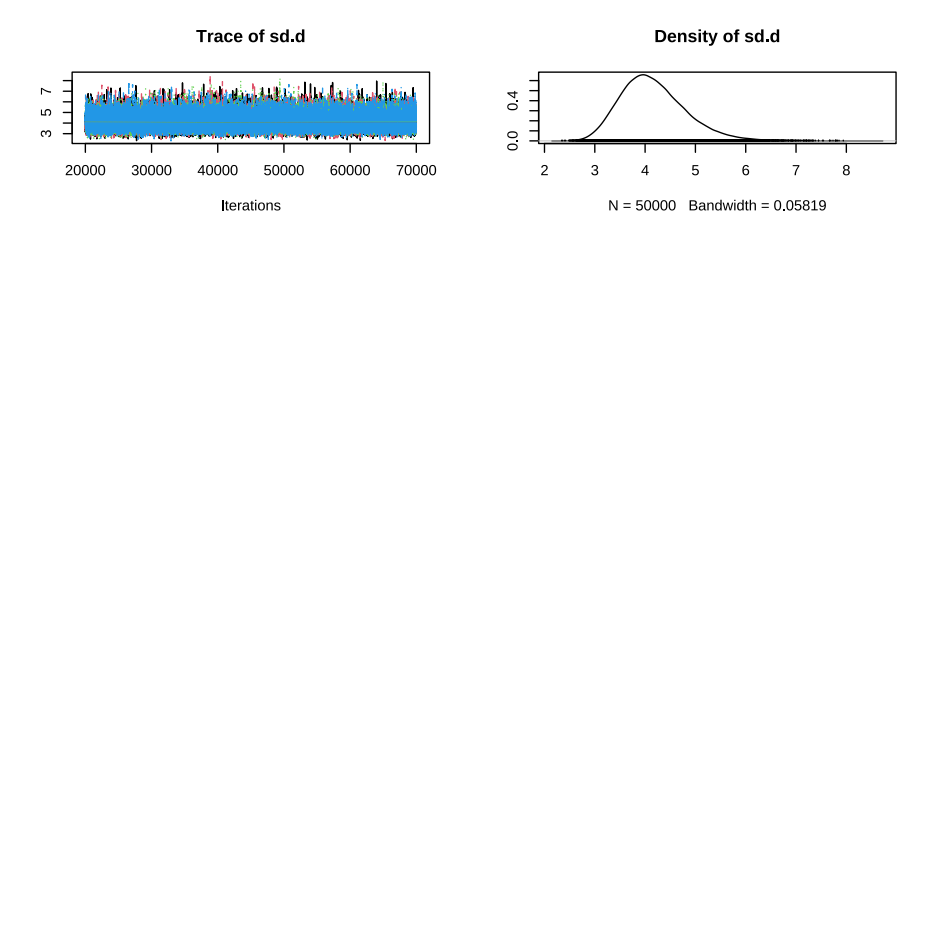


**Figure 2** **Trajectory diagram and density diagram: BBS score**

**
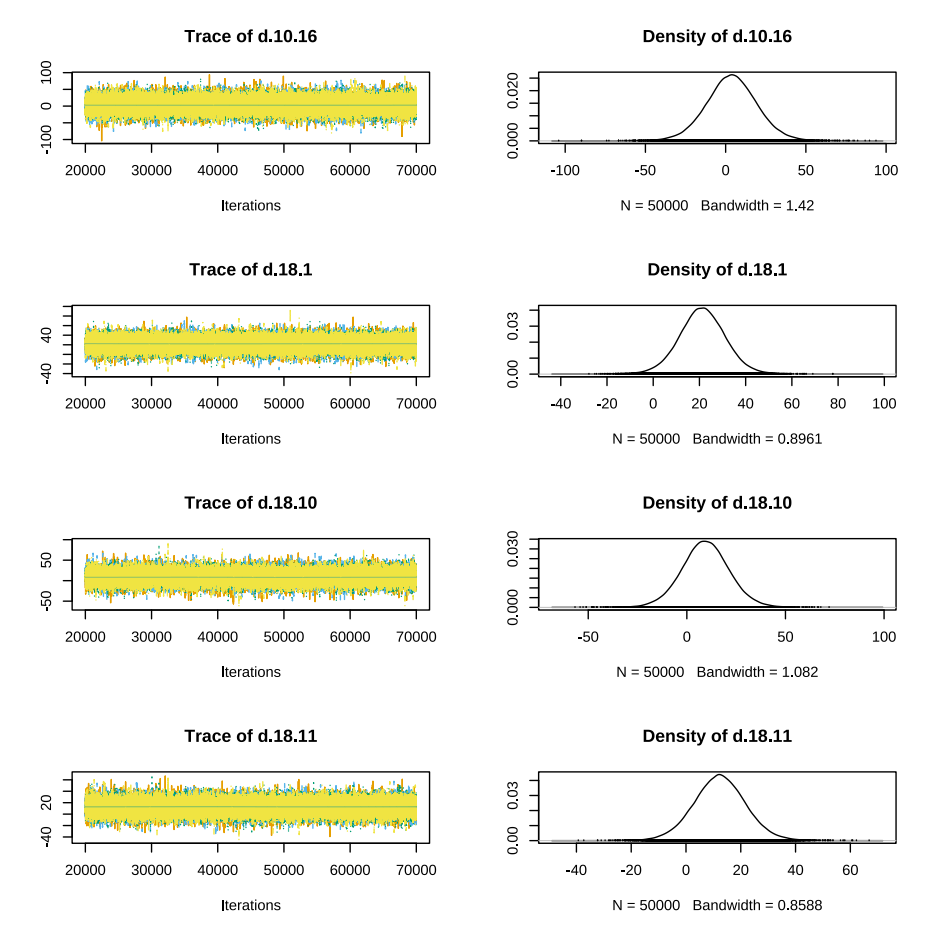
**


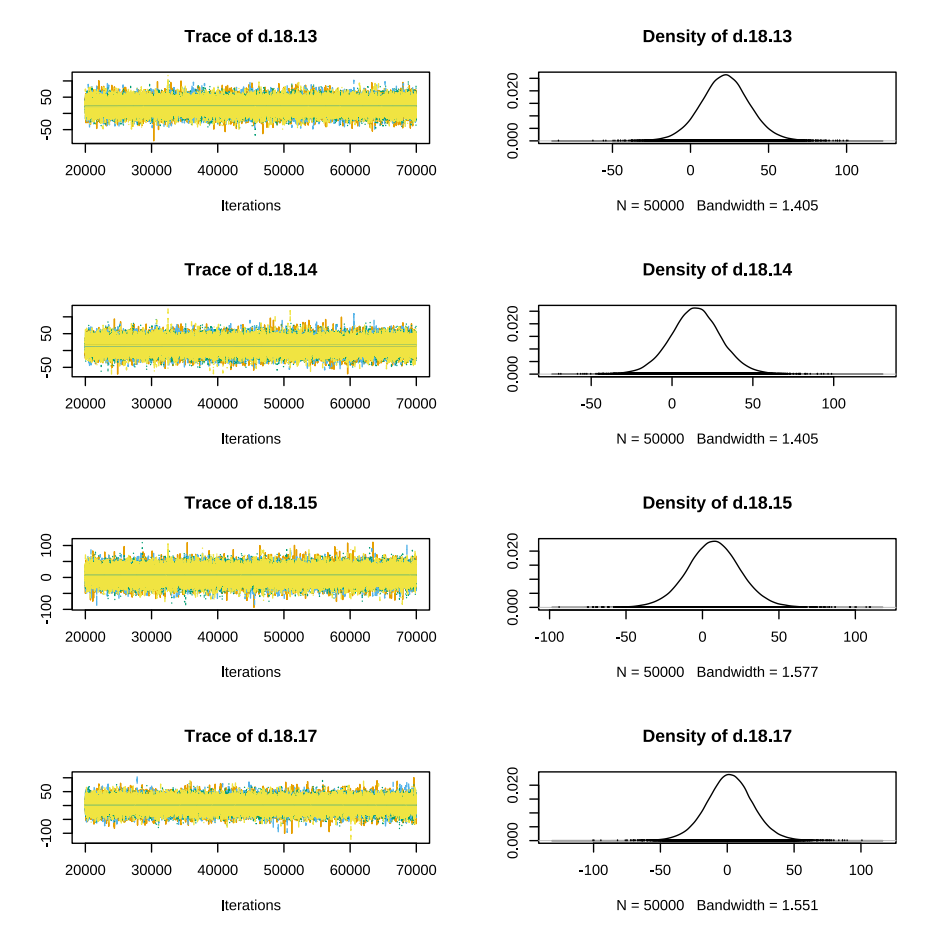

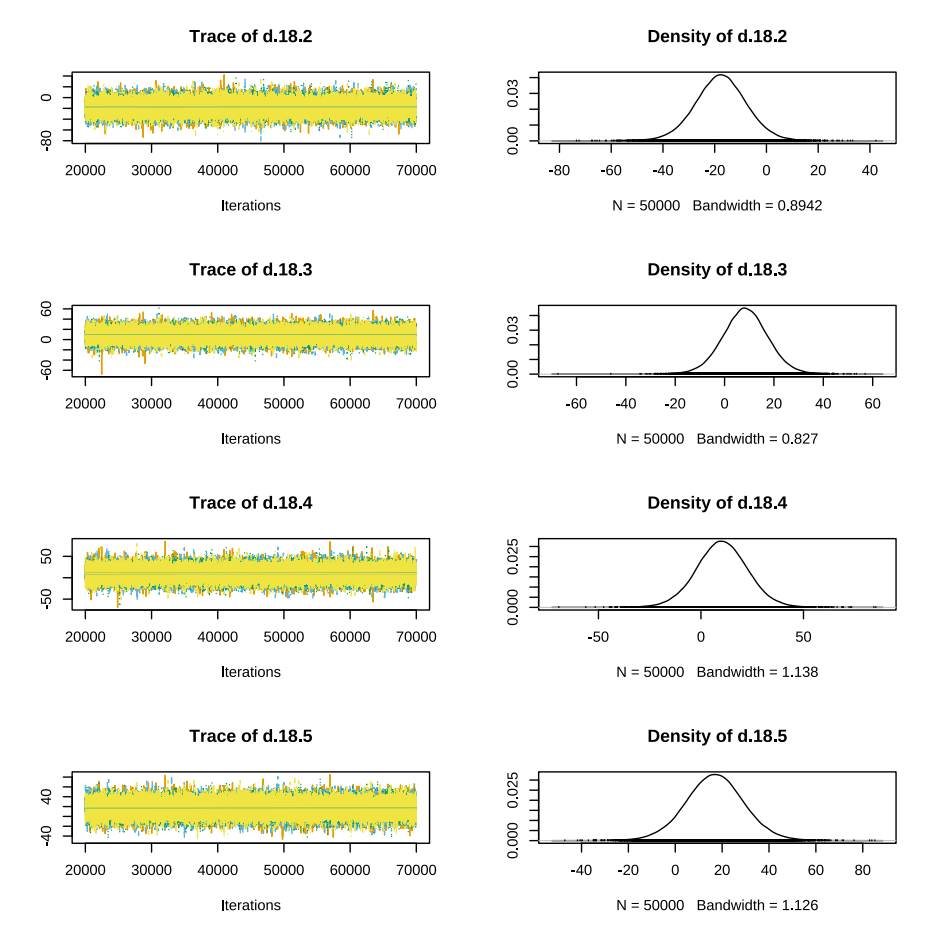

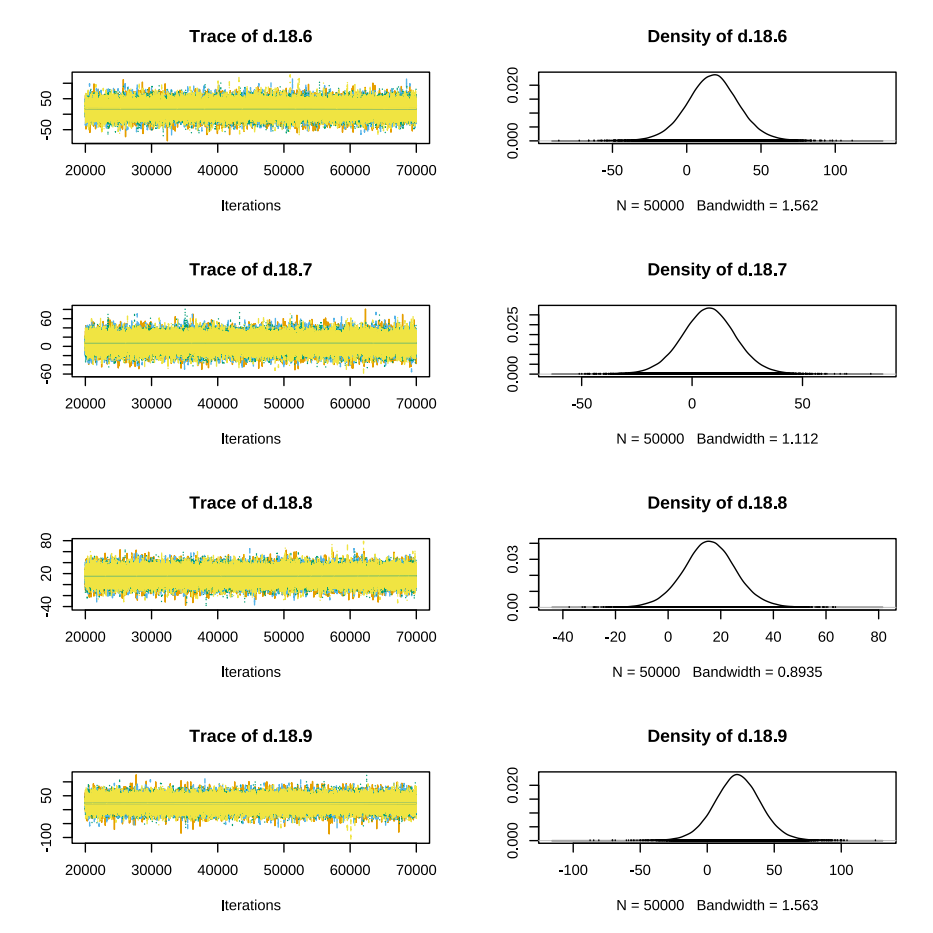

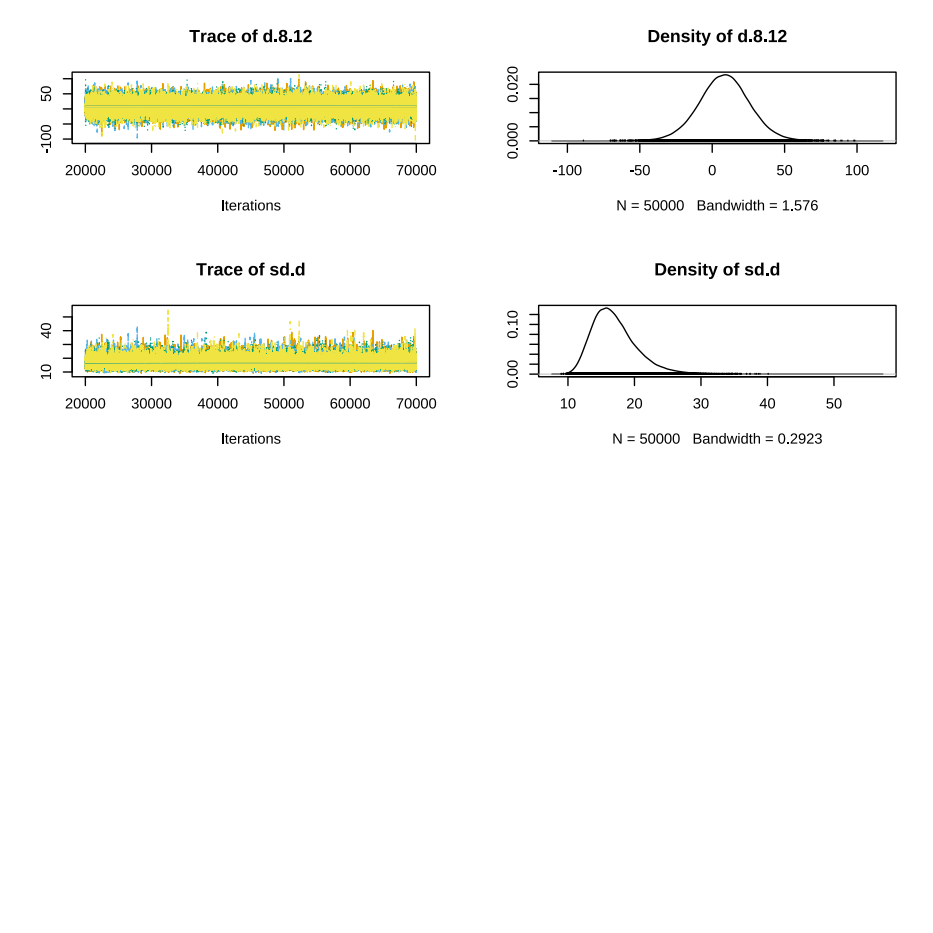


**Figure 3** **Trajectory diagram and density diagram: MBI score**

**
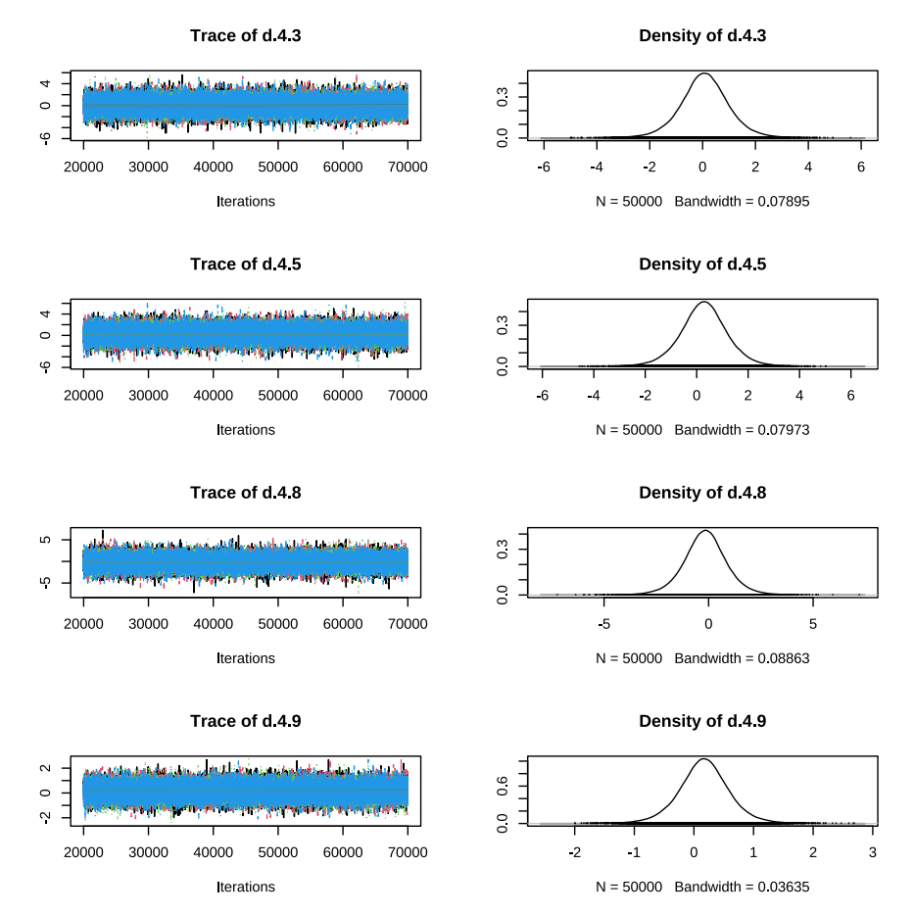

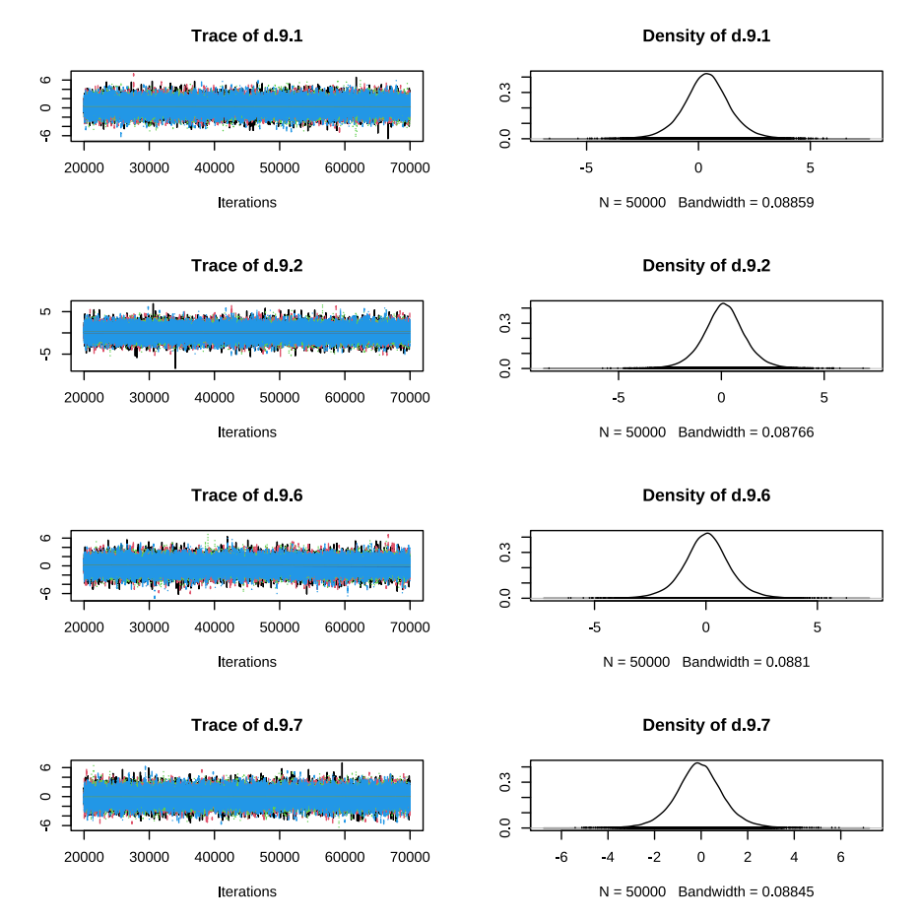

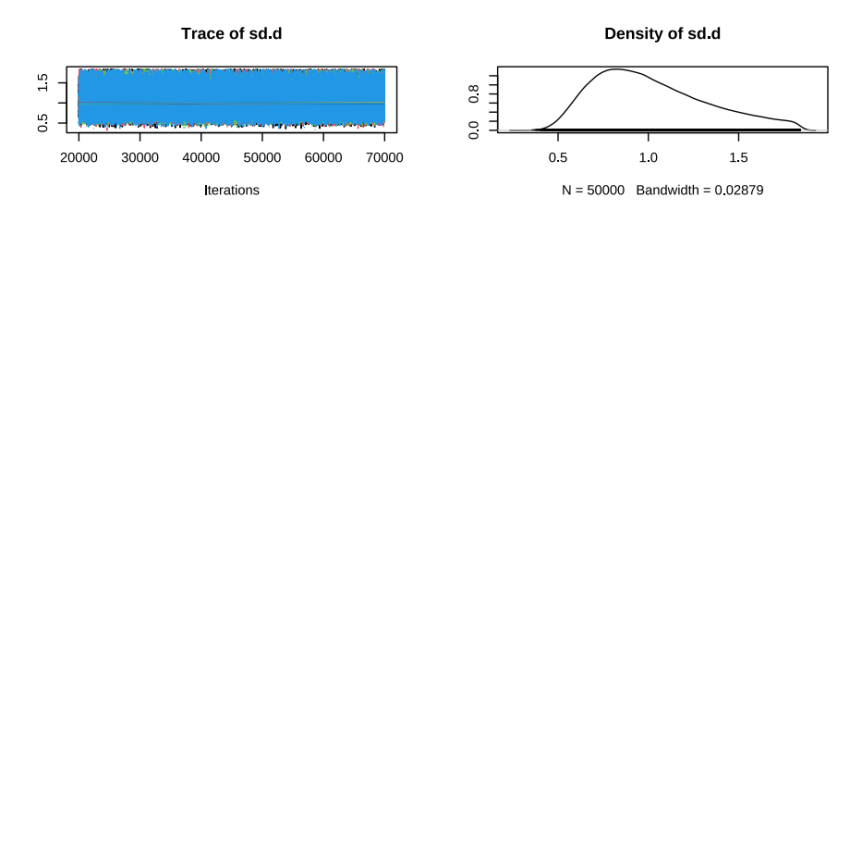
**

**Figure 4** **Trajectory diagram and density diagram: 10MWT**

**
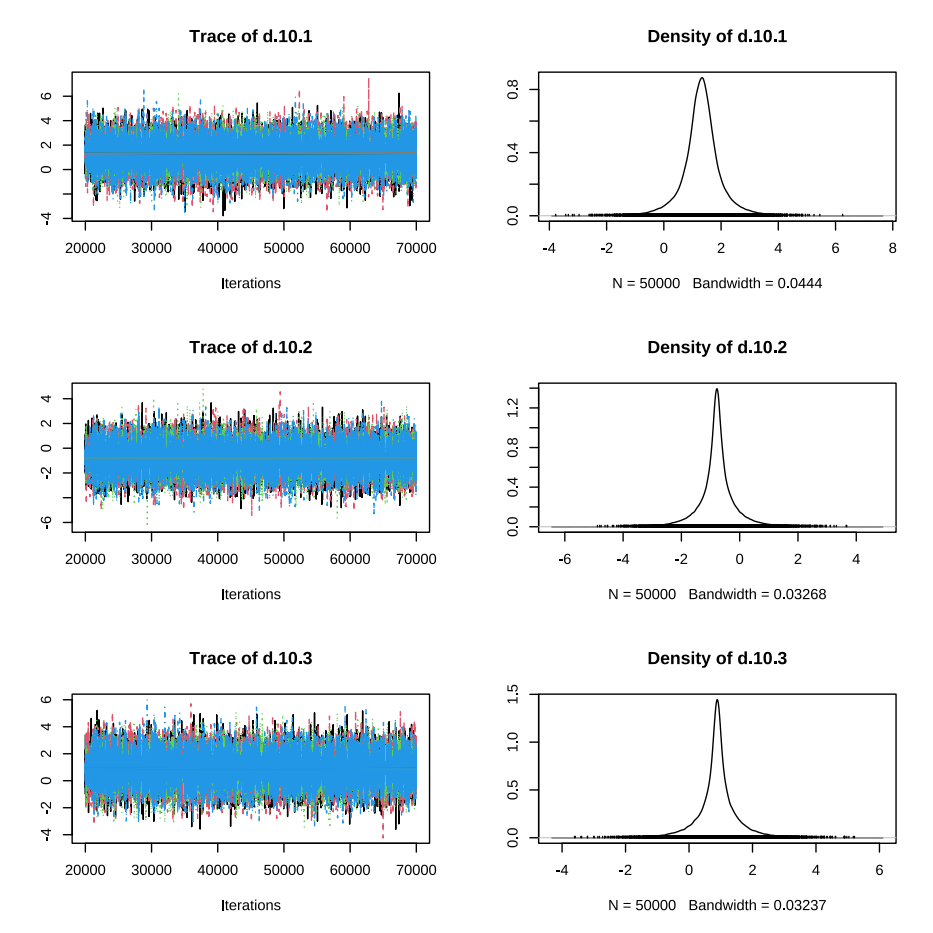
**


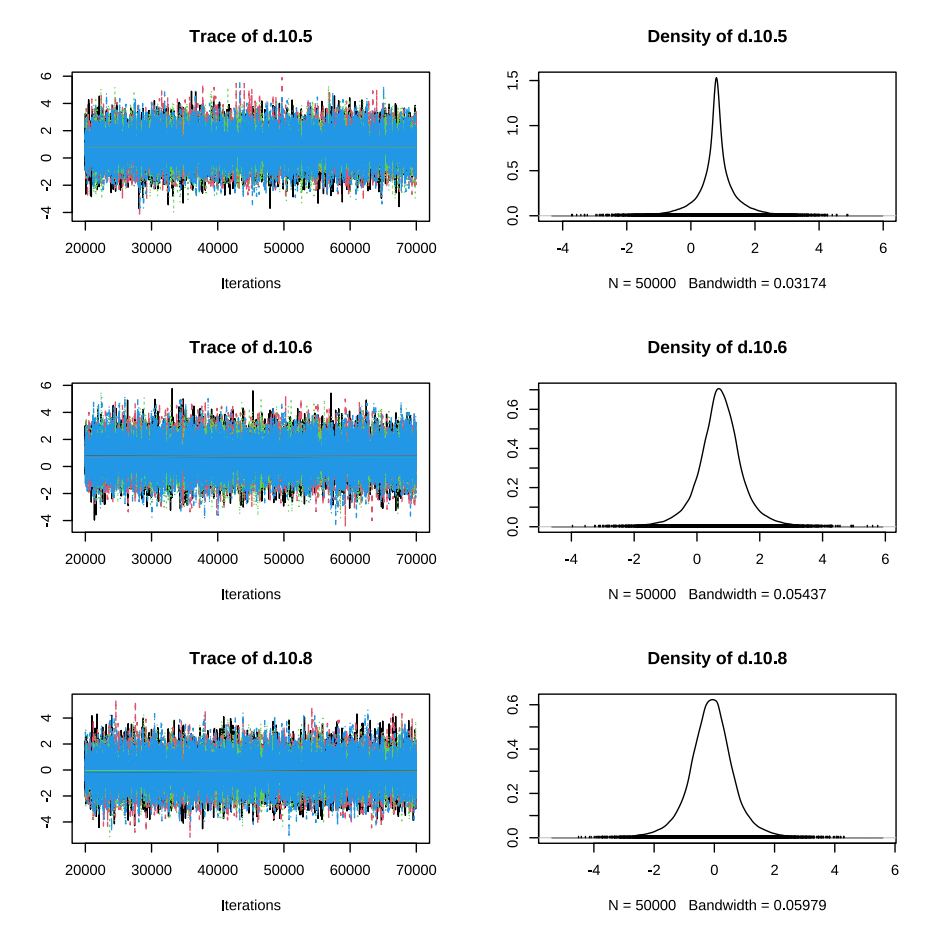


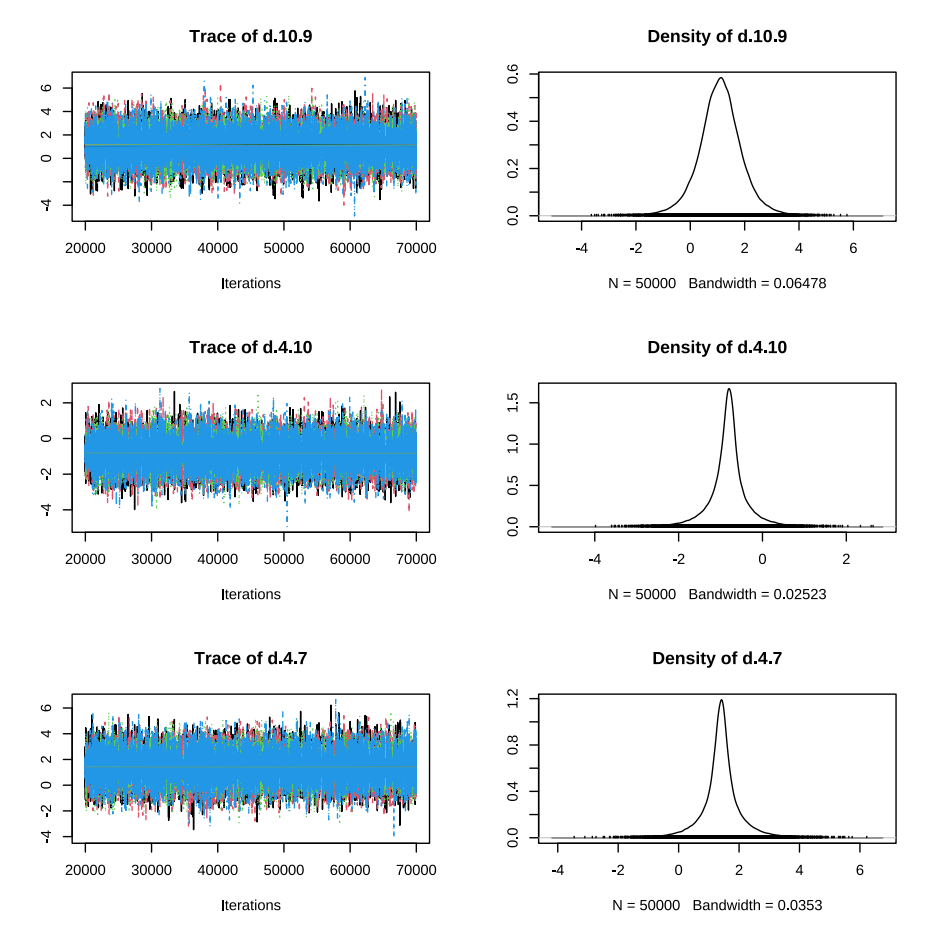

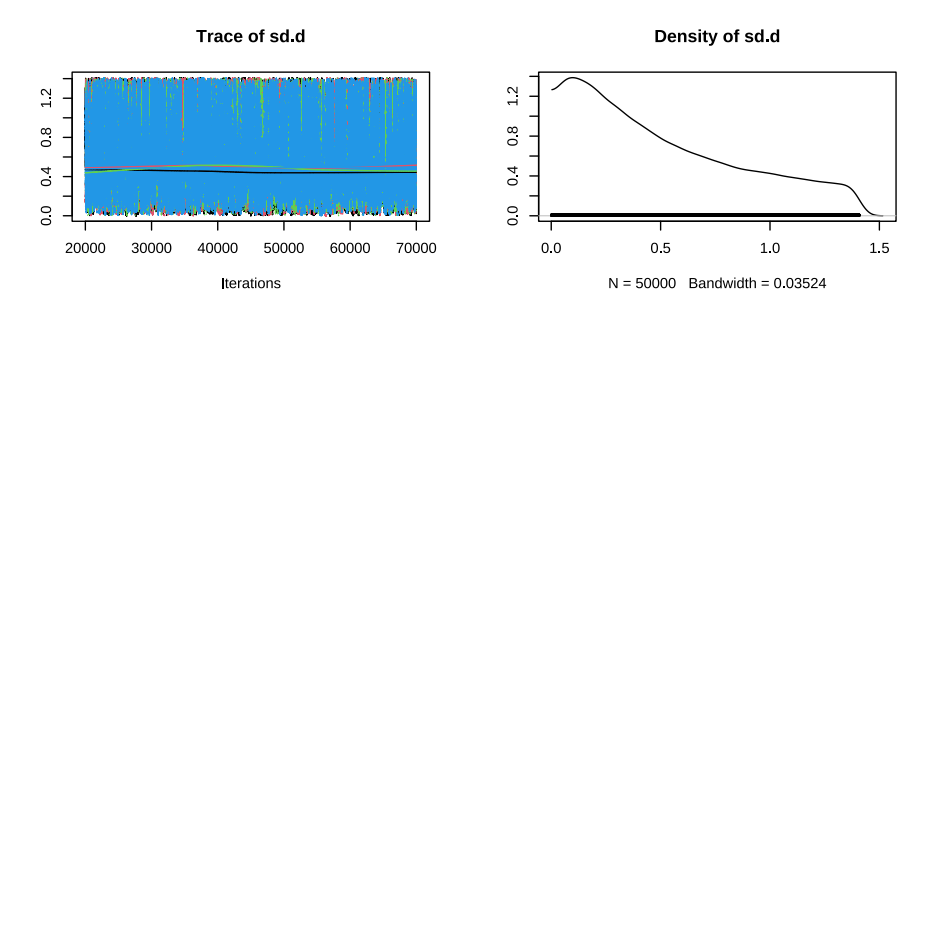


**Figure 5 Trajectory diagram and density diagram: FAC score**
