# Supplementary material for: Network meta-analysis of different electrical stimulation therapies for lower limb functional rehabilitation in stroke patients
Source: Front Neurol. 2026 Jan 12;16:1682671. doi: 10.3389/fneur.2025.1682671 (PMC12833568; doi:10.3389/fneur.2025.1682671)
Supplement: Supplementary file 5 [file Table_5.docx]

**Supplement**

diagnostic graph


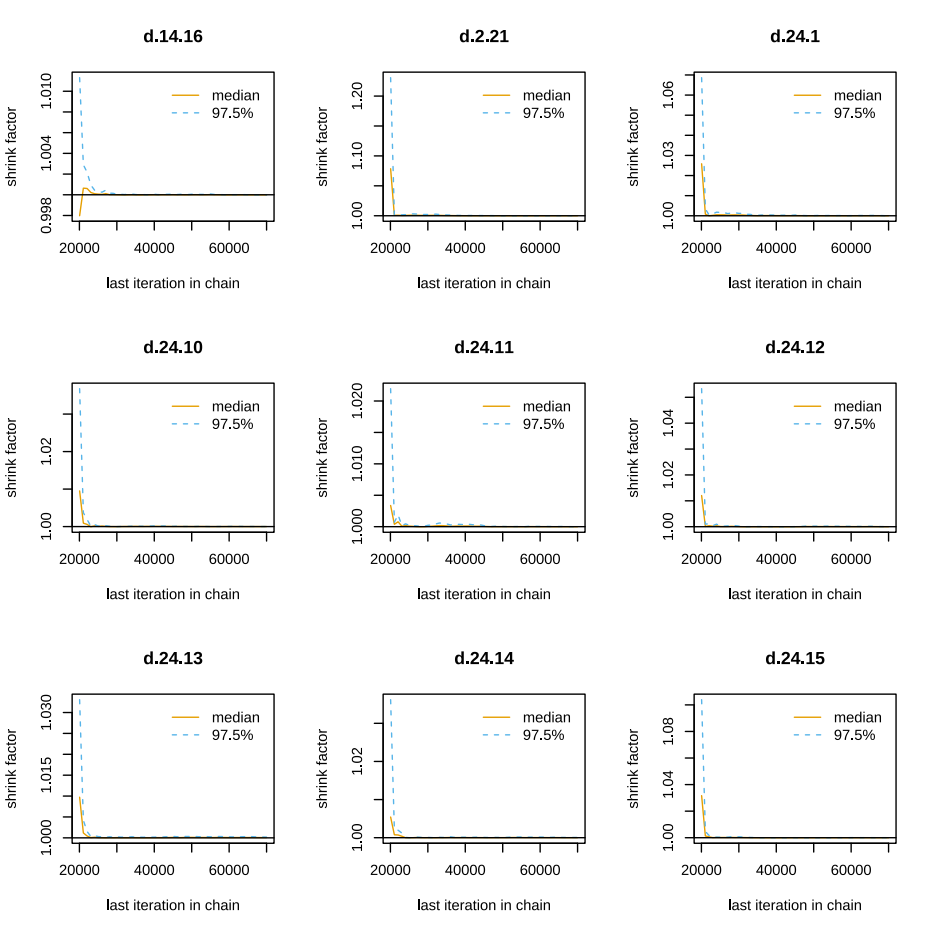

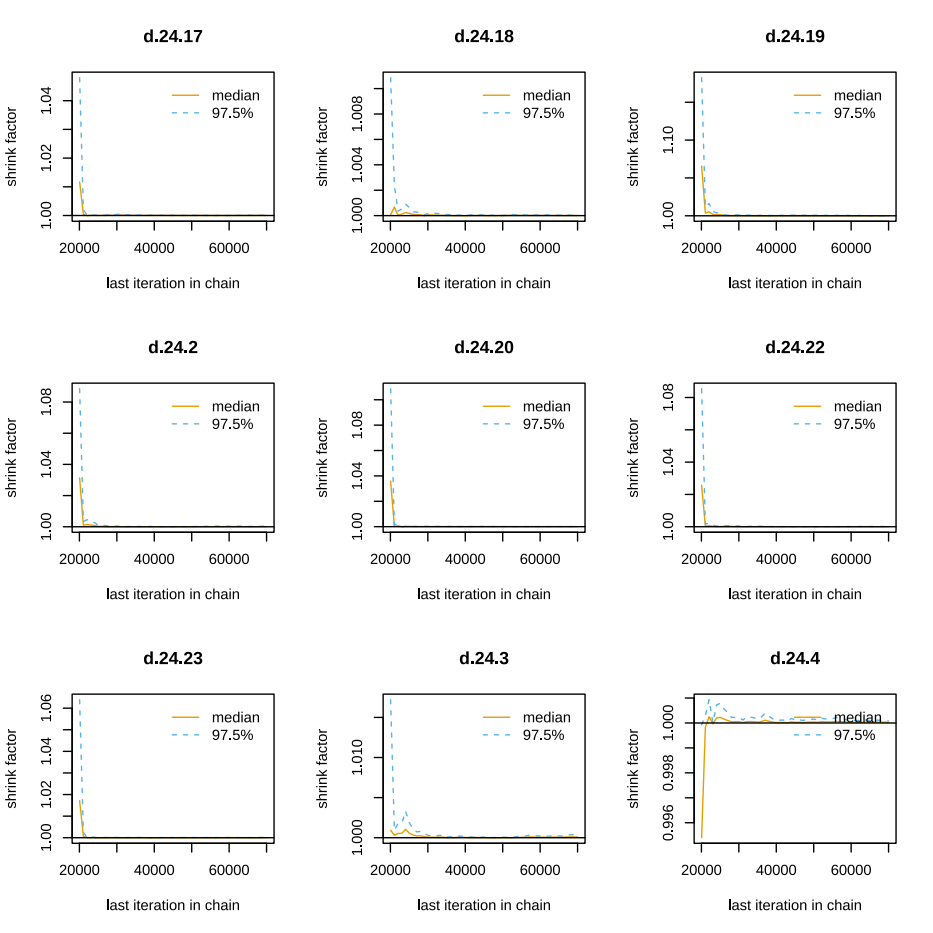

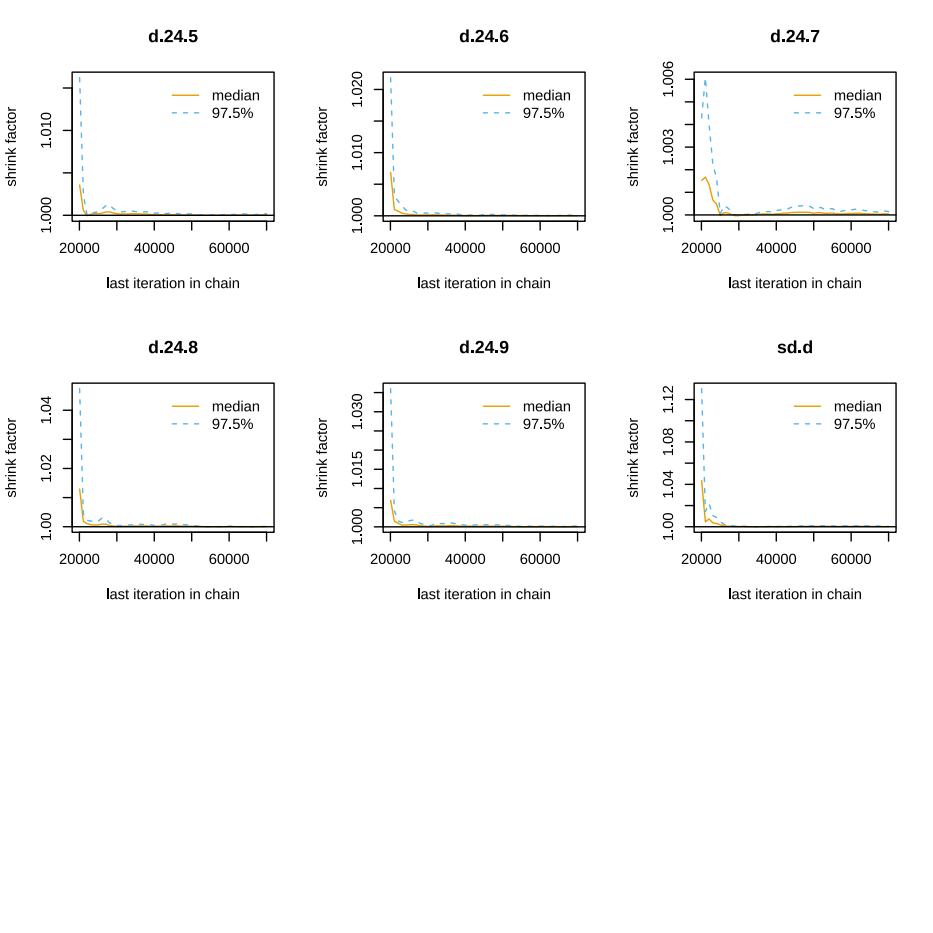


**Figure 1 diagnostic graph: FMA-L score**

**
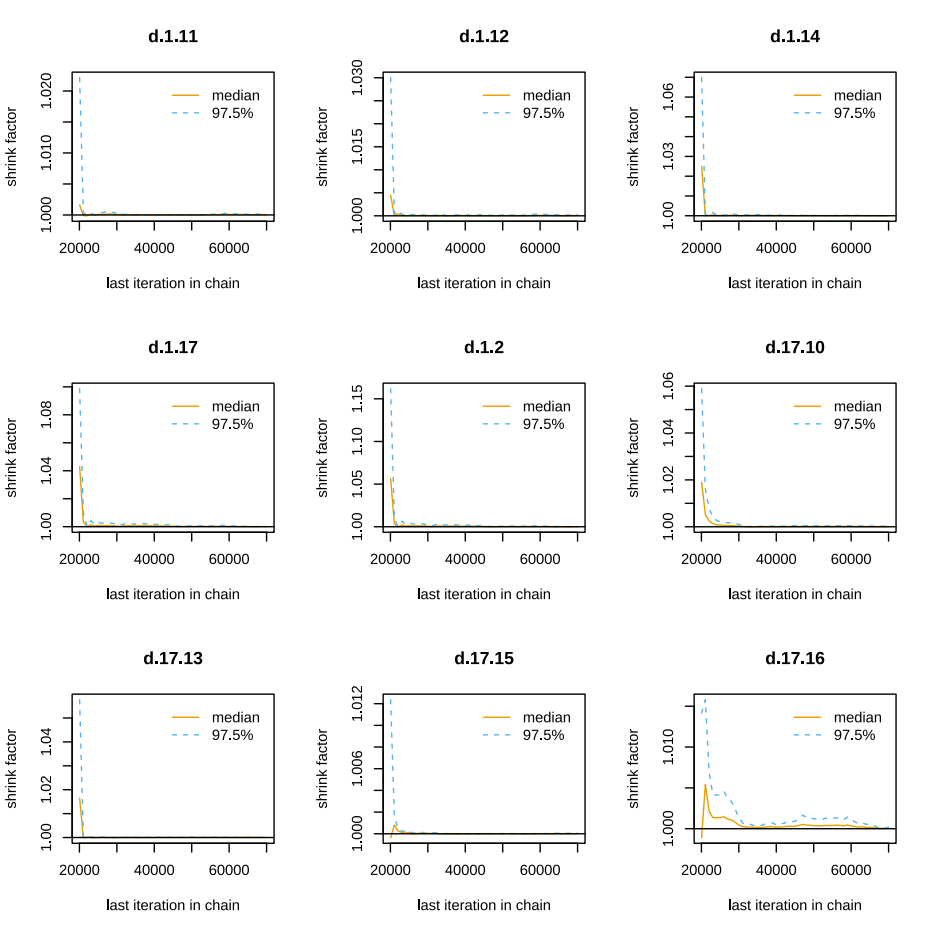

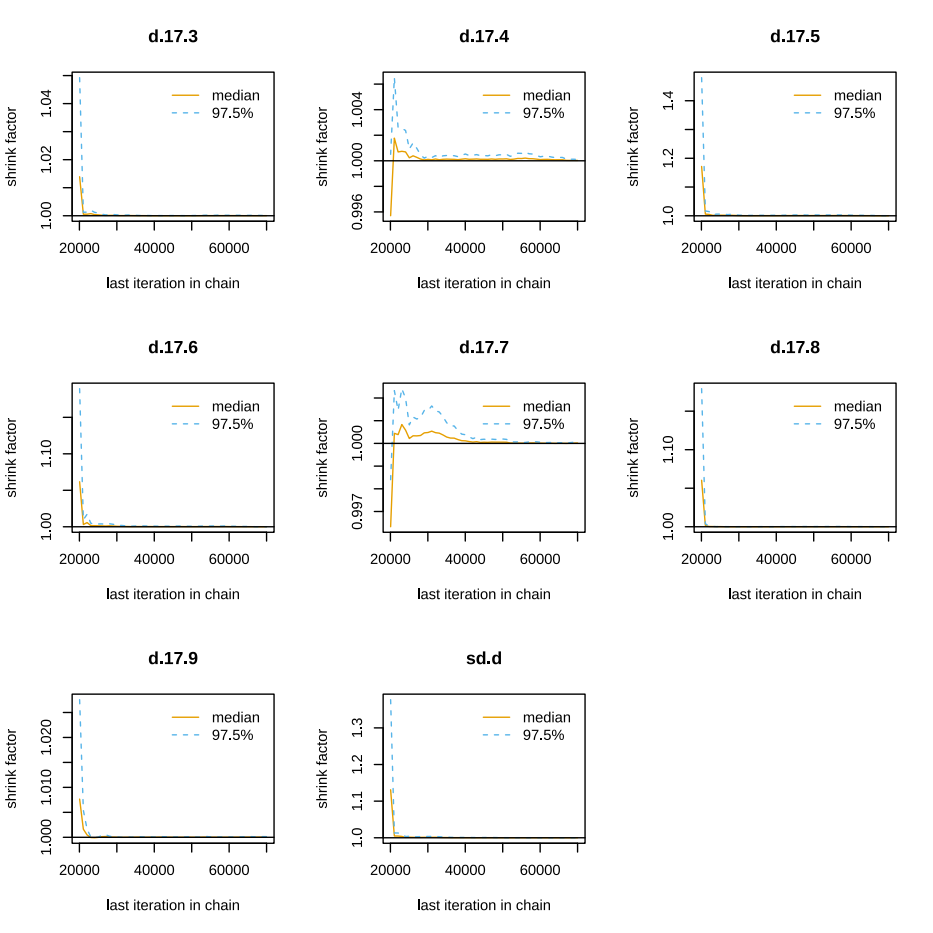
**

**Figure 2 diagnostic graph: BBS score**

**
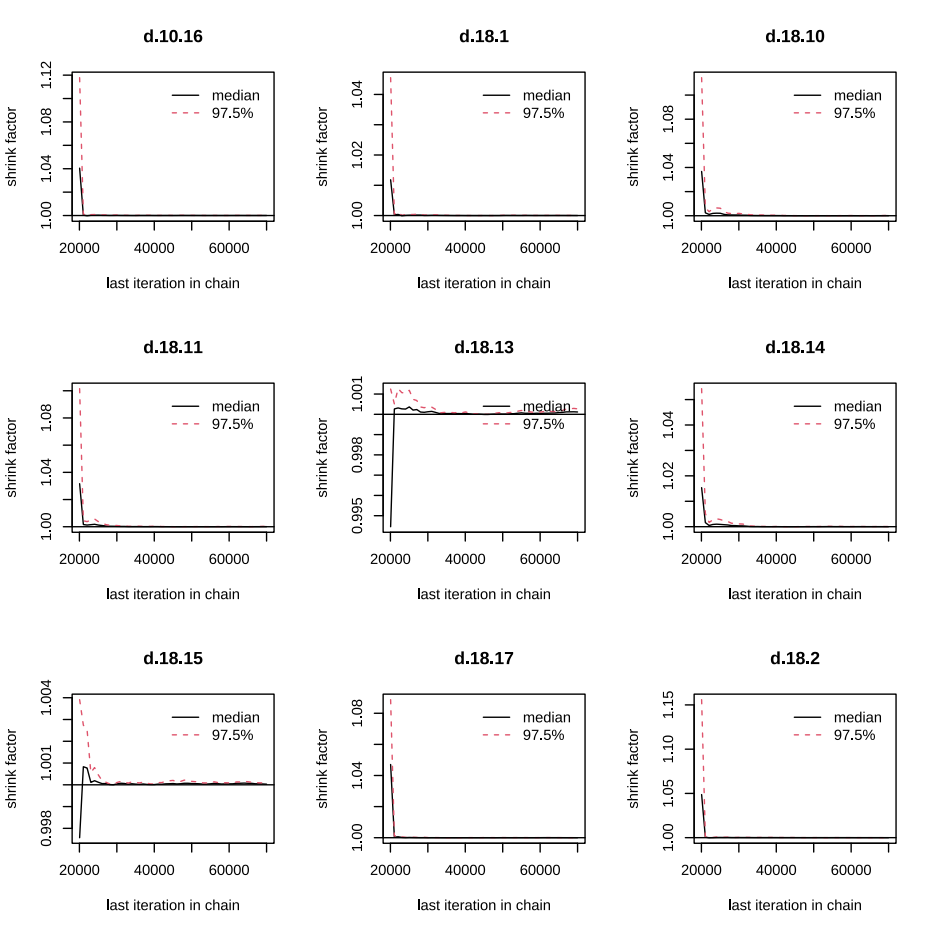

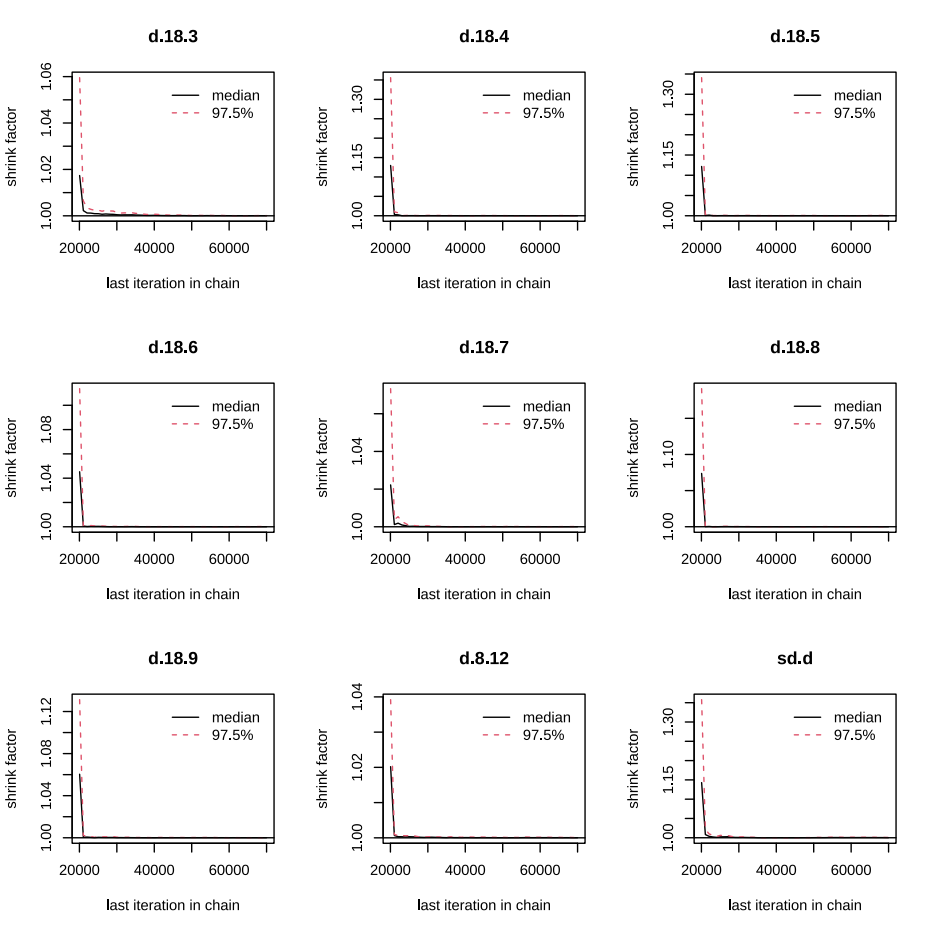
**

**Figure 3** **Trajectory diagram and density diagram: MBI score**

**
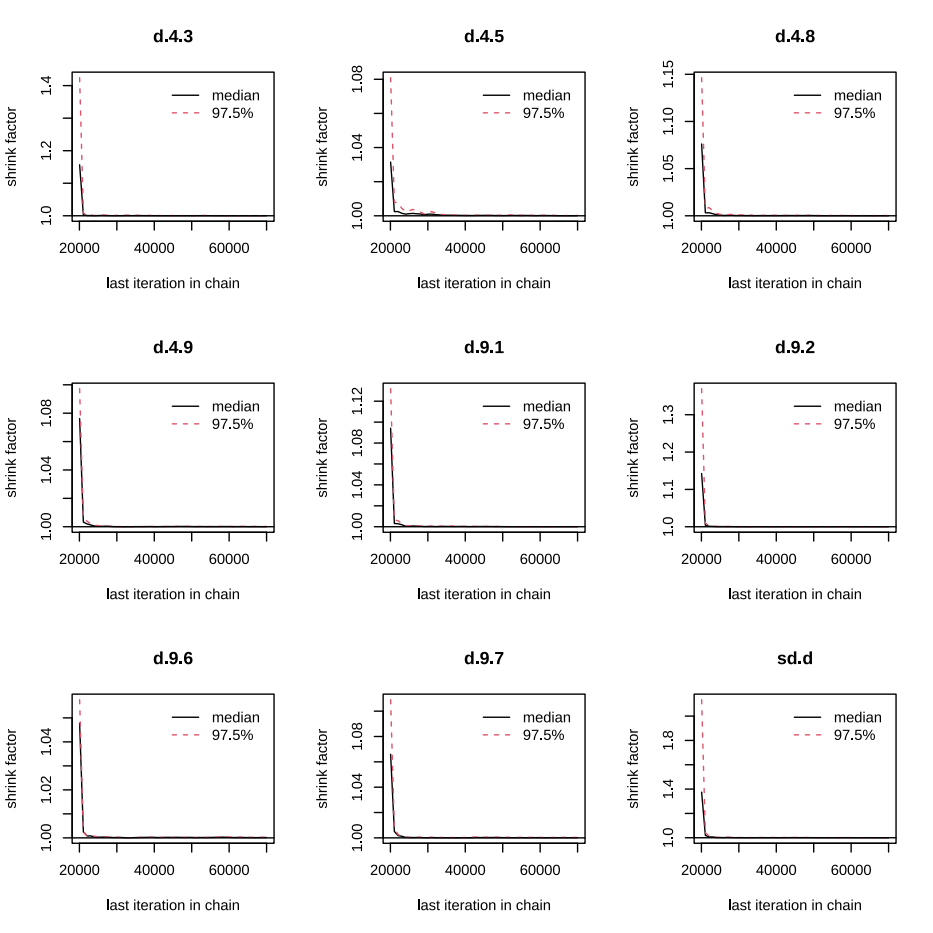
**

**Figure 4** **Trajectory diagram and density diagram: 10MWT**

**
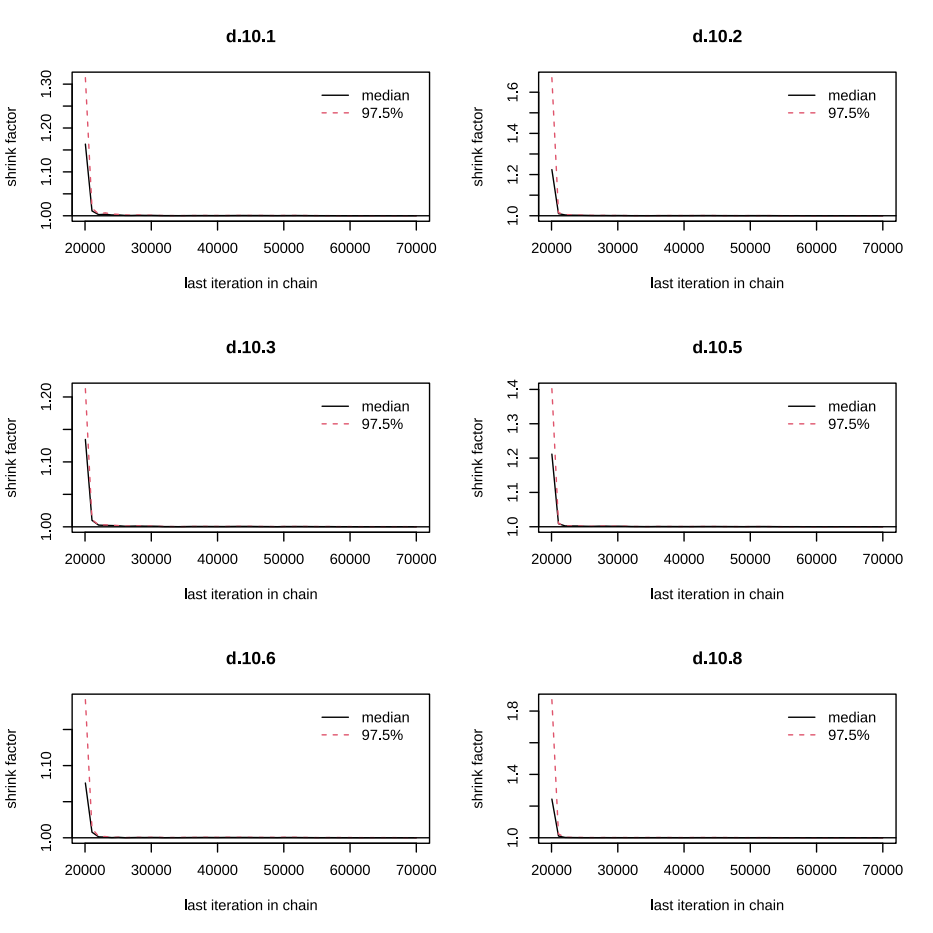
**


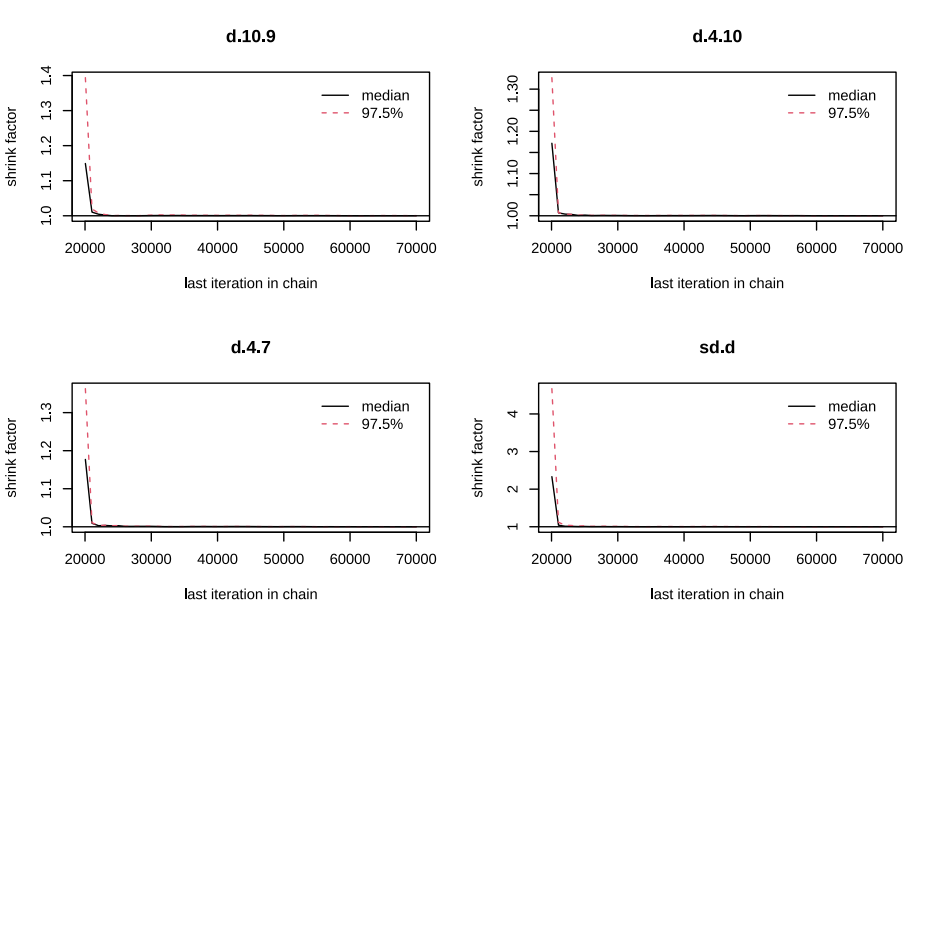


**Figure 4** **Trajectory diagram and density diagram: 10MWT**
